# ATP-Driven Remodeling of the Linker Domain in the Dynein Motor

**DOI:** 10.1016/j.str.2012.07.003

**Published:** 2012-10-10

**Authors:** Anthony J. Roberts, Bara Malkova, Matt L. Walker, Hitoshi Sakakibara, Naoki Numata, Takahide Kon, Reiko Ohkura, Thomas A. Edwards, Peter J. Knight, Kazuo Sutoh, Kazuhiro Oiwa, Stan A. Burgess

**Affiliations:** 1 Astbury Centre for Structural Molecular Biology, Institute of Molecular & Cellular Biology, Faculty of Biological Sciences, University of Leeds, Leeds LS2 9JT, UK; 2 MLW Consulting, 11 Race Hill, Launceston, Cornwall PL15 9BB, UK; 3 National Institute of Information and Communications Technology, Advanced ICT Research Institute, 588-2 Iwaoka, Nishi-ku, Kobe 651-2492, Japan; 4 Department of Life Sciences, Graduate School of Arts and Sciences, University of Tokyo, Komaba 3-8-1, Tokyo 153-8902, Japan

## Abstract

Dynein ATPases are the largest known cytoskeletal motors and perform critical functions in cells: carrying cargo along microtubules in the cytoplasm and powering flagellar beating. Dyneins are members of the AAA+ superfamily of ring-shaped enzymes, but how they harness this architecture to produce movement is poorly understood. Here, we have used cryo-EM to determine 3D maps of native flagellar dynein-c and a cytoplasmic dynein motor domain in different nucleotide states. The structures show key sites of conformational change within the AAA+ ring and a large rearrangement of the “linker” domain, involving a hinge near its middle. Analysis of a mutant in which the linker “undocks” from the ring indicates that linker remodeling requires energy that is supplied by interactions with the AAA+ modules. Fitting the dynein-c structures into flagellar tomograms suggests how this mechanism could drive sliding between microtubules, and also has implications for cytoplasmic cargo transport.

## Graphical abstract

**Graphical Abstract:**

**Figure:**
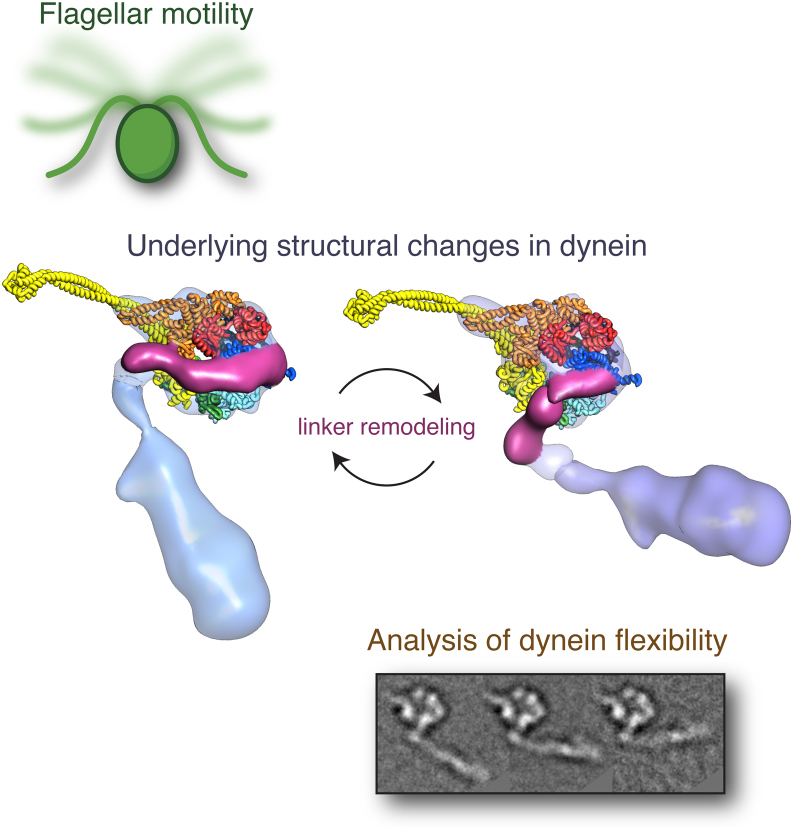

**Highlights:**

► Structural changes in axonemal and cytoplasmic dynein motors seen by cryo-EM ► Movement is amplified by a hinge action within dynein's linker domain ► The linker is a stable structure that is actively remodeled by dynein's AAA+ modules ► Dynein's neck subdomain interacts with AAA4 and is an intrinsic site of flexibility

## Teaser

Dyneins are AAA+ motor proteins that power both microtubule-based transport and flagellar motility. Now, Roberts et al. present cryo-EM structures of both classes of dynein, revealing nucleotide-induced bending and indicating that dynein generates movement by using the AAA+ ring to actively remodel its own linker.

## Introduction

Dyneins are a family of ATP-fueled motor proteins that move toward the minus ends of microtubules (Höök and Vallee, 2006). Cytoplasmic dynein transports diverse intracellular cargoes, generates forces at multiple sites within the cell division machinery, and is implicated in several forms of neurological disorder (Karki and Holzbaur, 1999). A closely related dynein is involved in intraflagellar transport (Scholey, 2008). In contrast, axonemal dyneins power the beating of cilia and flagella (Gibbons, 1981). Axonemal dynein dysfunctions cause ciliopathies resulting in infertility and improper left-right body asymmetry (Fliegauf et al., 2007). The mechanism of dynein, and thus the molecular basis for all these forms of movement, is only beginning to emerge.

**Figure 1:**
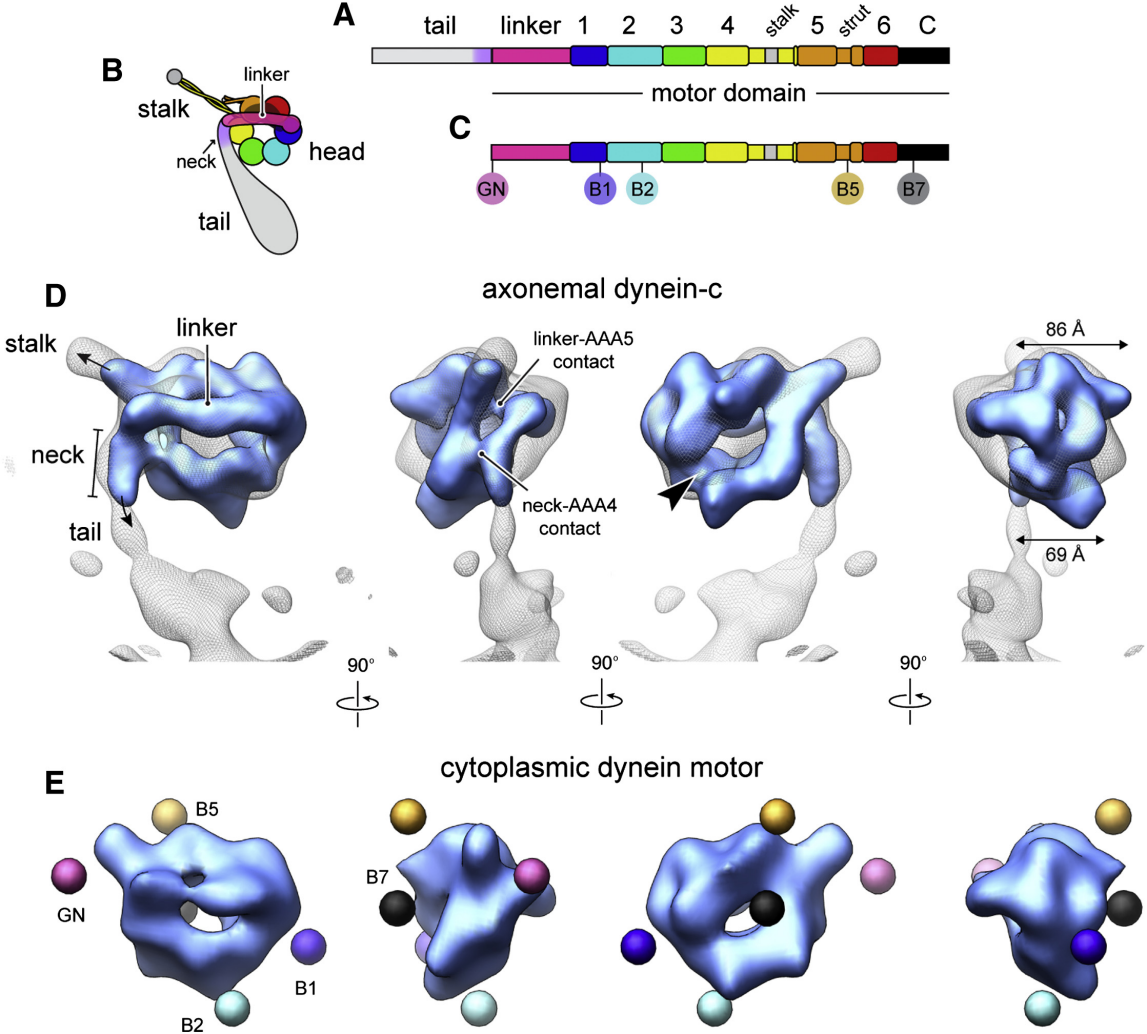
Architecture of the Head Domains of Axonemal Dynein-c and Cytoplasmic Dynein (A) Sequence diagram of the dynein-c heavy chain from *C. reinhardtii*. The six AAA+ modules are numbered (1–6). (B) Schematic of dynein domain organization (colored as in A). (C) Sequence diagram of the 380 kDa cytoplasmic dynein motor domain from *D. discoideum*. Insertion sites of a GFP tag at the N terminus (GN) and four BFP tags previously engineered within the head (B1, B2, B5, and B7) are indicated (Roberts et al., 2009). (D) Cryo-EM reconstruction of the head domain of dynein-c prepared in the absence of nucleotide (blue isosurface) with the variance map (gray wire mesh) overlaid. The resolution of the map is 19 Å according to the 0.5 FSC (Fourier shell correlation) criterion (Figure S1A). The arrowhead indicates the gap in the ring between AAA1 and AAA2. (E) Cryo-EM reconstruction of the head domain of cytoplasmic dynein in the presence of ADP (blue isosurface). The resolution of the map is 23 Å according to the 0.5 FSC criterion (Figure S1D). Colored spheres depict the mean locations of tags (shown in C) inserted in the motor domain, as derived in Figure S2.

To perform their functions, dyneins have a divergent tail domain that specifies distinct cargo-binding and oligomerization properties, attached to a motor domain of 300–400 kDa. High-resolution structural information is available for the motor domain of cytoplasmic dynein (Carter et al., 2011; Kon et al., 2011, 2012; Schmidt et al., 2012), building on earlier insights from electron microscopy (EM) and sequence analysis (Burgess et al., 2003; Gee et al., 1997; Neuwald et al., 1999; Roberts et al., 2009; Samsó et al., 1998). The head domain contains six AAA+ modules, which fold into a ring (Figures 1A and 1B). The first module (AAA1) is the main site of ATP hydrolysis, whereas AAA2 and AAA3–AAA4 appear to be subsidiary nucleotide-binding and hydrolysis sites, respectively. N terminal to AAA1 is a linker domain that connects to the tail. Dynein's microtubule-binding domain lies at the tip of a long coiled-coil stalk that protrudes as an extension from AAA4. The stalk interacts with a second coiled-coil strut embedded in AAA5 (also known as the buttress). The microtubule-binding domain and AAA1 are coupled by a long-range communication pathway (Carter et al., 2008; Gibbons et al., 2005; Kon et al., 2009), allowing dynein to sequentially bind and release its microtubule track during cycles of ATP hydrolysis.

The linker domain is an essential component of the dynein motor. First discovered in a monomeric axonemal dynein (dynein-c) from *C. reinhardtii* (Burgess et al., 2003), the linker forms the linkage between the tail and AAA1. It has an elongated structure comprising five subdomains and arches over the AAA+ ring (Kon et al., 2012). In the available crystal structures of *D. discoideum* and *S. cerevisiae* cytoplasmic dynein, the position of the linker differs subtly with respect to the ring (Kon et al., 2012; Schmidt et al., 2012). Whether this stems from differences in nucleotide occupancy, protein engineering, crystal packing, or source organism is unclear. Importantly, previous FRET and two-dimensional (2D) EM studies have suggested that the linker undergoes a large-scale “priming” motion across the ring in response to ATP binding at AAA1 (Burgess et al., 2003; Kon et al., 2005; Roberts et al., 2009). Microtubule binding is thought to trigger the reverse movement, in which the linker delivers a force-generating powerstroke. A cleft has recently been identified within the unprimed linker, leading to the hypothesis that a hinge exists at this site (Kon et al., 2012; Schmidt et al., 2012). Crucially, however, a lack of structural information on the primed state of dynein means that whether hinging actually occurs within the linker is unknown. Because dynein is distantly related to AAA+ proteins that use the energy of ATP binding and hydrolysis to remodel or dismantle substrates, it is possible that the linker might be reshaped in an analogous manner, but direct evidence is lacking.

Insights into dynein architecture in situ have come from EM of the axoneme. Within the cylindrical bundle of microtubule doublets in the axoneme, the inner-arm dyneins (including dynein-c) form cross-bridges between adjacent doublets and repeat at 96 nm intervals along their length (Goodenough and Heuser, 1985). Cryo-electron tomography (cryo-ET) has produced 3D maps of the axonemal repeat (Bui et al., 2008, 2009; Heuser et al., 2009; Movassagh et al., 2010; Nicastro et al., 2005, 2006). Images of mutant axonemes lacking single dynein species have allowed assignment of densities to individual dyneins, so that the location of dynein-c within the tomogram is known (Bui et al., 2008). Thus, there is now the opportunity to correlate structures of isolated dynein-c with its structures in situ.

In this work we present cryo-EM structures of dynein-c and the cytoplasmic dynein motor in different nucleotide states, including the primed conformation. The structures reveal critical sites of conformational change in the AAA+ ring and a hinge action within the linker. Analysis of an engineered dynein shows that the linker is an intrinsically stable domain, indicating that hinging is actively promoted by interactions with the AAA+ modules. Applying image classification to our cryo-EM data provides insights into dynein's flexibility and dynamics that would be difficult to establish by other methods. Finally, we build complete models of dynein-c and dock these into tomograms of the axoneme, suggesting how linker remodeling is harnessed to drive microtubule sliding in cilia and flagella.

## Results

### Cryo-EM Structure of Axonemal Dynein-c

We began by determining the structure of native dynein-c purified from *C. reinhardtii* flagella (Sakakibara et al., 1999) in the absence of nucleotide at ∼20 Å resolution using cryo-EM and image processing (Figure 1D; see Figure S1 available online). To aid alignment of the head domain, the tail and stalk domains of the molecule were masked out during refinement because these domains are flexibly attached to the head (Figure 6C; see also Burgess et al., 2003). A variance map provides a good indication of the tail and stalk locations (Figure 1D), as we found in earlier 2D studies (Burgess et al., 2004b). We also determined the cryo-EM structure of the cytoplasmic dynein motor domain from *D. discoideum* (Kon et al., 2004) lacking the tail, for comparison (Figure 1E). The cryo-EM maps account for all described 2D views of the head (Figures S1B and S1E cf. S1G). This indicates that dynein motor structure was well preserved in earlier negative-stain studies and allows previously mapped positions of GFP-based tags to be related to the 3D volumes (Figure S2).

The head domain of dynein-c comprises an asymmetric ring-like structure with the linker domain arching over one face (Figure 1D). The overall architecture is conserved with cytoplasmic dynein (Figure 1E). However, in the dynein-c map there is additional density that continues beyond the end of the linker, which we assign as the “neck” subdomain of dynein's tail (Burgess et al., 2003). There is a striking (∼90°) kink between the linker and neck. An important consequence of this sharp kink is to direct the path of the tail away from the stalk, thereby preventing a steric clash with the microtubule.

**Figure 2:**
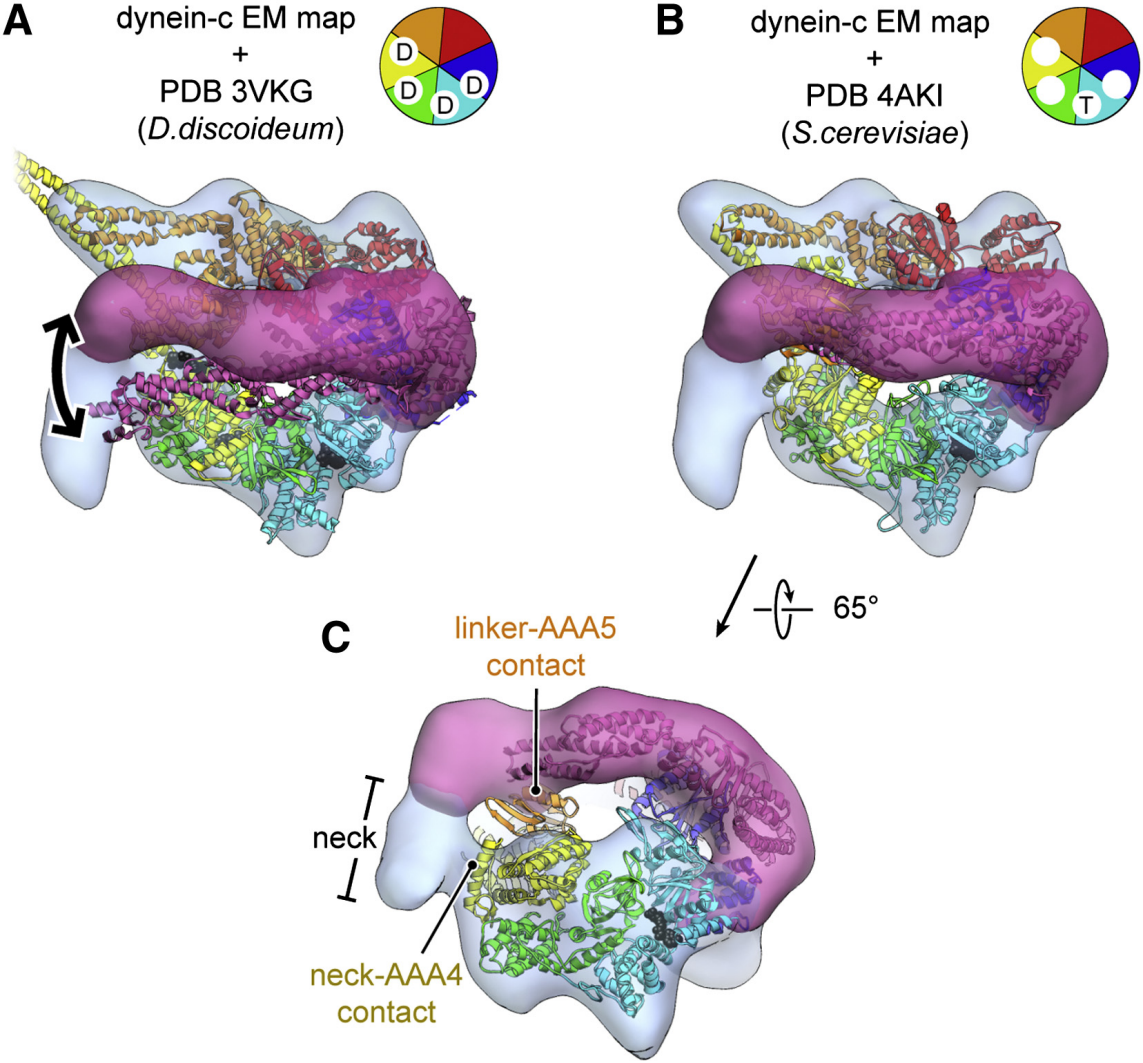
Crystal Structure Docking and Neck-AAA4 Interaction (A) Ribbon representation of the *D. discoideum* cytoplasmic dynein crystal structure in the presence of ADP (PDB 3VKG; Kon et al., 2012) docked into the *C. reinhardtii* axonemal dynein-c cryo-EM map prepared in the absence of nucleotide (transparent surface). Arrow depicts the difference in linker position between these structures obtained in different nucleotide states. (B) Docking of the *S. cerevisiae* cytoplasmic dynein crystal structure prepared in the absence of nucleotide (PDB 4AKI; Schmidt et al., 2012). Schematics show the nucleotide occupancy in the crystal structures (D, ADP; T, ATP). (C) Rotated view, showing the interaction between the linker and AAA5 and a novel contact between the neck subdomain of the tail and AAA4.

The fit of the available cytoplasmic dynein crystal structures into the axonemal dynein-c cryo-EM map is generally very satisfactory, although there are notable differences (Figures 2A and 2B). Quantitative comparison shows that the dynein-c map obtained in the absence of nucleotide is closer to the *S. cerevisiae* crystal structures with no nucleotide in AAA1 (e.g., PDB 4AKI), compared to the *D. discoideum* structure with ADP bound (PDB 3VKH; cross-correlation scores 0.84 and 0.76, respectively) (Kon et al., 2012; Schmidt et al., 2012). Similarities between the dynein-c and *S. cerevisiae* structures include a large gap in the ring between AAA1 and AAA2 (Figure 1D, arrowhead), which originates from the AAA1 nucleotide-binding pocket being in a more open conformation than the ADP structure. This suggests that an open nucleotide-binding pocket at AAA1 is an intrinsic feature of dynein structure in the absence of nucleotide.

### Linker-AAA5 and Neck-AAA4 Interactions

The linker domain of dynein-c forms an interaction with AAA5 in the ring (Figure 2C). This contact strongly resembles that seen in the crystal structure of *S. cerevisiae* dynein involving AAA5's helix 2 and β hairpin insert (Carter et al., 2011). In the *D. discoideum* ADP crystal structure (Kon et al., 2012), the linker is slightly offset and lies over AAA4 instead of AAA5 (Figure 2A, arrow). These data support the notion that the linker undergoes a small movement between ADP and no-nucleotide states, from AAA4 to AAA5.

The dynein-c map also shows an unanticipated contact between the ring and the neck subdomain of the tail (Figure 2C). After the sharp kink, the neck interacts with AAA4, appearing to contact the N-terminal helical insert of AAA4 based upon crystal structure fitting. This neck-AAA4 contact is a stronger feature in the cryo-EM map than the linker-AAA5 contact, persisting at thresholds up to 4.2 σ from the mean density, compared to 2.6 σ for the linker contact. This suggests that in native dynein-c, two contacts with the ring must be broken in order for the linker to undergo its large-scale priming motion: one involving the linker itself, and the other involving the neck.

### Cryo-EM Structures of the Head Domain in the Primed State

**Figure 3:**
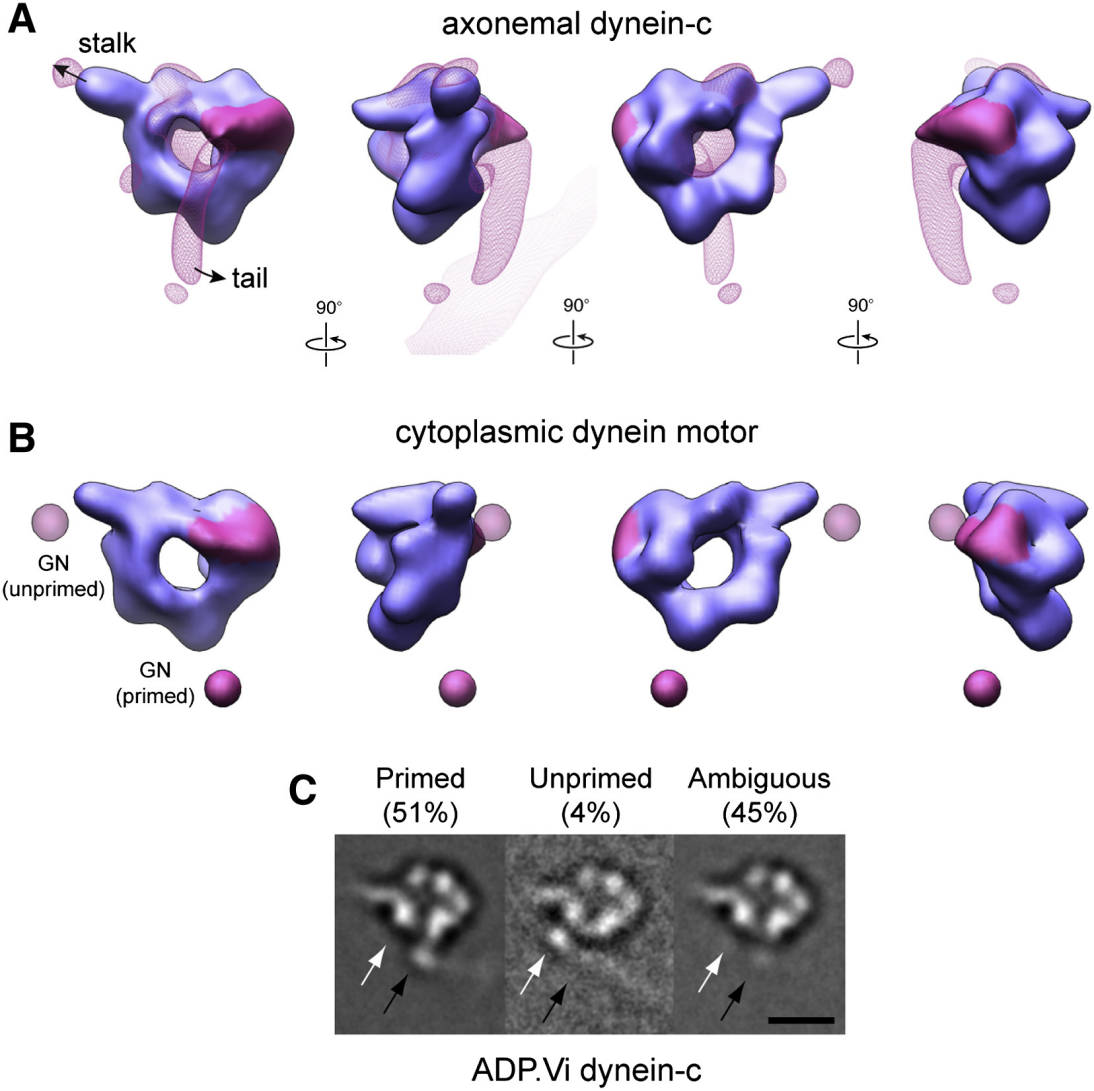
Architecture of the Primed Dynein Head (A) Cryo-EM reconstruction of the dynein-c head in the ADP.Vi state, with the variance map overlaid (magenta wire mesh). The base of the linker is colored magenta. The resolution of the map is 22 Å according to the 0.5 FSC criterion (Figure S1A). (B) Cryo-EM reconstruction of the cytoplasmic dynein head, bearing a E2027Q substitution, in the ATP state. Mean primed and unprimed locations of a GFP tag on the motor N terminus are indicated (solid and faded magenta spheres, respectively) (Roberts et al., 2009). The resolution of the map is 25 Å according to the 0.5 FSC criterion (Figure S1D). (C) Cryo-EM class averages of ADP.Vi-dynein-c showing variation in the site of tail emergence from the head (black arrows, primed position; white arrows, unprimed position). Scale bar is 10 nm.

To obtain insights into dynein's motile mechanism, we determined cryo-EM maps of axonemal and cytoplasmic dynein heads in the primed conformation (Figure 3). Primed molecules were generated using two methods. Axonemal dynein-c was treated with ATP and vanadate (Vi) to trap ADP.Vi-dynein. For the cytoplasmic dynein motor, we introduced a Walker B substitution into AAA1 (E2027Q) that allows ATP binding but prevents its hydrolysis. FRET assays indicate that both ADP.Vi-dynein and ATP-dynein (E2027Q) adopt a primed conformation (Kon et al., 2005).

**Figure 4:**
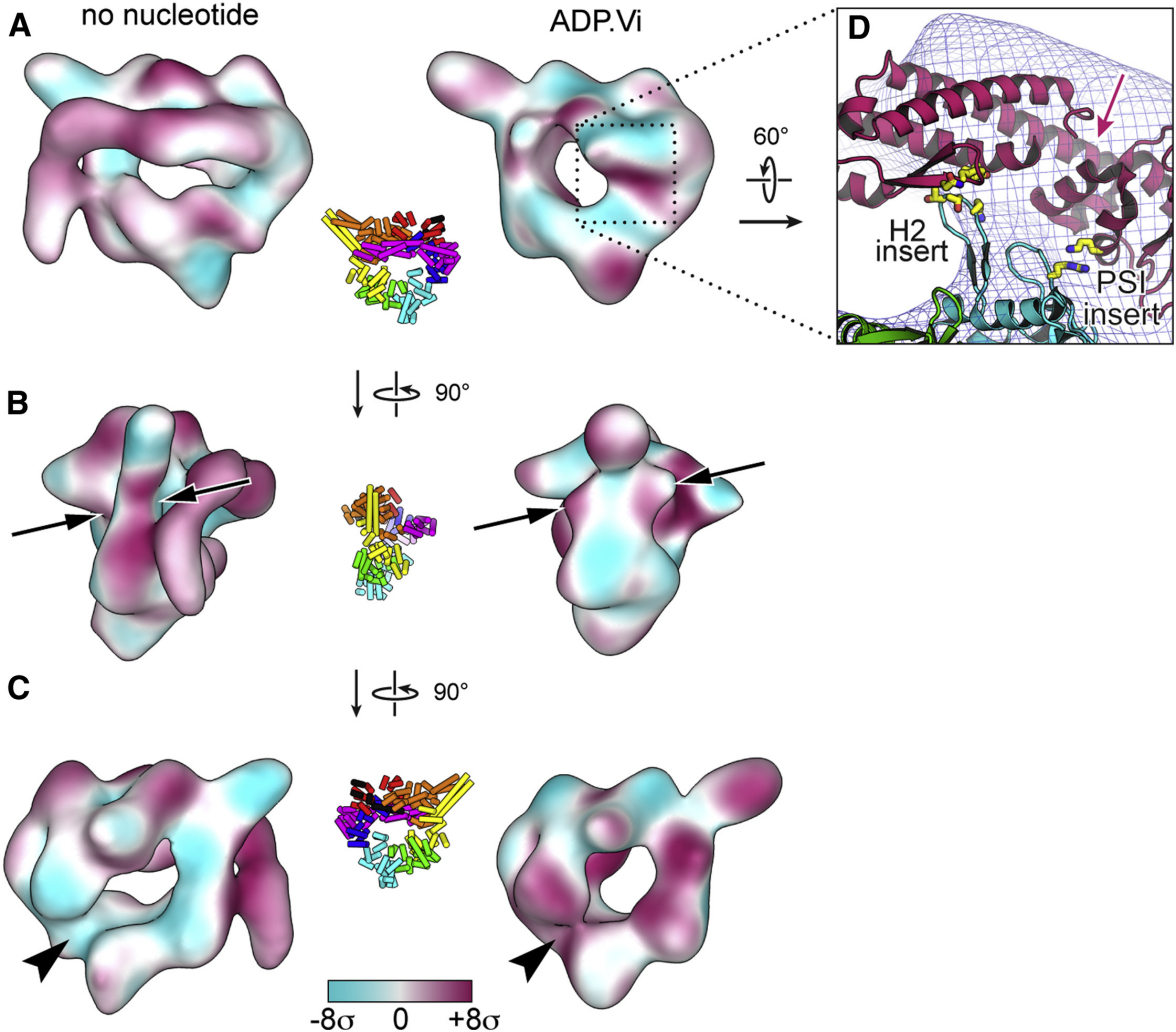
Nucleotide-Driven Structural Changes (A–C) Three views showing changes in density between dynein-c heads prepared in the absence of nucleotide and with ADP.Vi bound. Maps were filtered to the same resolution (22 Å), normalized to a mean voxel intensity of 0 and σ of 1, aligned, and subtracted pairwise from each other. Voxel intensities in the resulting difference maps were used to color the surface of each map, according to the chart. Maroon indicates a positive change in density from the other structure, whereas cyan indicates a negative change. Insets show corresponding views of *S. cerevisiae* cytoplasmic dynein crystal structure colored as in Figure 1. Changes at the linker contact site (B, arrows) and between AAA1 and AAA2 (C, arrowheads) are indicated. (D) Enlargement showing the *D. discoideum* dynein crystal structure (PDB 3VKG) (Kon et al., 2012) docked into the ADP.Vi-dynein-c map (wire mesh). Interacting residues between the linker and the β hairpin inserts in AAA2 in the crystal structure are shown (yellow sticks). The single α helix spanning the cleft in the linker is indicated with an arrow.

The primed head structures (Figures 3A and 3B) show pronounced differences in sections of the ring and the linker compared to their unprimed counterparts. Differences between the dynein-c EM maps are shown in Figure 4. The large gap in the ring between AAA1 and AAA2 (Figure 4C, arrowheads) closes in the primed state, consistent with nucleotide binding between these modules. As a result of this closure, the primed ring appears more circular. The ring also shows a marked change near the linker contact site at AAA4 and AAA5 (Figure 4B, arrows). This is likely to correlate with the differences seen in the stalk/strut region in the primed molecule, although these elements remain in close proximity as seen by negative-stain EM (Figure S3B, arrows).

The most striking change in the linker of primed molecules is that the distal segment of the linker is missing from the density maps (Figure 3). Sorting individual cryo-EM images of dynein-c based on the position of the tail as it emerges from the head indicates that this lack of linker density is due to variability in its position relative to the AAA+ ring (Figure 3C). Most frequently, the tail emerges in a primed position (Figure 3C, black arrows), but occasionally, it emerges in the unprimed position close to the stalk (Figure 3C, white arrows). These observations suggest that in ADP.Vi-dynein the distal linker adopts two preferred positions, but is predominantly in a primed position, displaced across the ring.

The path of the primed linker across the ring is suggested by a variance map of ADP.Vi-dynein-c molecules, in which a cylindrical feature extends from the linker's base (Figure 3A). This path shows strong agreement with the site of tail emergence from the primed molecule (Figure 3C). It is also compatible with the location of a GFP tag attached to the end of the primed linker in cytoplasmic dynein (Figure 3B, magenta sphere), indicating a similar primed linker position in both axonemal and cytoplasmic dyneins. The base subdomain of the linker appears largely unmoved from the unprimed conformation (Figure 3), indicating that the origin of linker displacement is a rotation of the distal segment relative to the base. The hinge site is close to a previously identified cleft between linker subdomains (Kon et al., 2012; Schmidt et al., 2012), spanned by a single α helix (Figure 4D). These EM data provide evidence that hinging within the linker domain provides the main source of mechanical amplification in dynein.

### Hinging within the Linker Is Promoted by Interactions with the AAA+ Ring

A hinge within the linker domain raises two conceptually different models for how the linker might amplify movement during dynein's ATPase cycle. First, the linker could be inherently flexible at the hinge site and explore different angles, which are fixed in place at particular times via contacts with the AAA+ modules. In this scenario, linker motion would be akin to a thermal ratchet, with directionality being provided by ATP-driven changes in the AAA+ modules. At the other extreme, the linker might be intrinsically stiff along its length and require energy from interactions with the AAA+ modules to promote “remodeling” at the hinge site. To our knowledge, it is not possible to distinguish between these models with existing data because crystal structures provide snapshots of fixed states of the linker rather than dynamic information, and the mechanical properties of the linker when it is detached from the ring are unknown.

**Figure 5:**
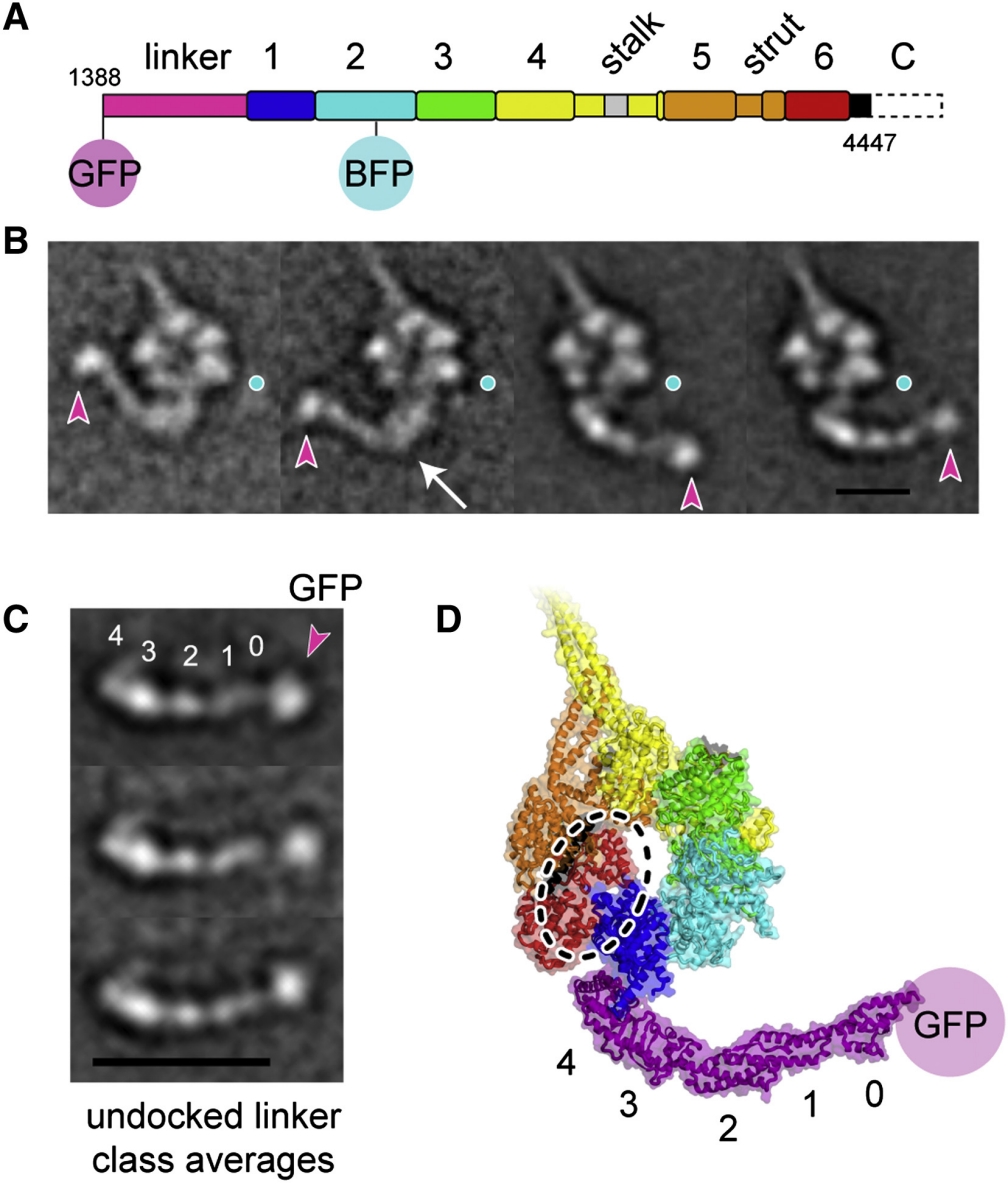
The Linker Is a Stable Domain when Undocked from the AAA+ Ring (A) Deletion of C-terminal amino acids 4,448–4,730 (dashed outline) from the cytoplasmic dynein motor yields a construct displaying frequent linker undocking in ADP. Analysis of the intact (i.e., linker docked) molecules is shown in Figure S3. Biochemical properties of this construct are described in Numata et al. (2011). The N-terminal tail domain is also deleted in this construct. (B) Negative-stain class averages showing the linker undocked from the AAA+ ring. The GFP tag at the end of the linker is marked with a magenta arrowhead. The global mean position of the BFP tag in AAA2 is overlaid in each class (cyan disc). A sharp bend in the linker is only occasionally present when it lies to the left of the ring (white arrow). Scale bar is 10 nm. See also Movie S1. (C) Class averages after alignment based on linker features showing that the linker is comparatively stiff along its length. The classes differ mainly in the position of GFP, which is flexibly attached to the linker. See also Movie S2. Five subdomains within the linker are numbered after Kon et al. (2012), and the GFP tag at the linker N terminus (GN) is indicated. Scale bar is 18 nm. See also Movie S2. (D) Model of a linker-undocked molecule derived from *D. discoideum* dynein crystal structure PDB 3VKG (Kon et al., 2012). The precise part of the structure that deforms to enable undocking is not clear but might involve the C-terminal end of the linker or the N-terminal part of AAA1. The location of the truncated C-terminal region is shown with a dashed outline.

We discovered conditions that induce the linker to frequently separate or “undock” from the AAA+ ring, allowing us to investigate these models. Our earlier work on a cytoplasmic dynein construct lacking the entire C-terminal region showed a subset of molecules in which the linker appeared to undock (Roberts et al., 2009), but the scarcity of these molecules precluded a detailed analysis. Now, we find that a construct with a smaller C-terminal truncation (Figure 5A) displays frequent linker undocking (∼66% of molecules) in ADP conditions (Figure 5B). This effect is nucleotide dependent because linker undocking does not occur in the presence of ATP and Vi (Figure S3B). These results show that although the C-terminal region lies on the opposite face of the ring from the linker, its truncation can destabilize interactions between the linker and the AAA+ modules.

The undocked linker adopts a range of positions relative to the AAA+ ring (Figure 5B). However, careful analysis shows that this is principally due to flexibility within a thin tether connecting the linker and ring (see Movie S1). Aligning the undocked linkers by single-particle methods shows that the domain itself is rather stiff along its length (Figure 5C; Movie S2). All five linker subdomains identified in the crystal structure (Figure 5D; Kon et al., 2012) are discernable when the linker is separated from the ring, suggesting that is a stably folded structural entity. Moreover, the shape of the undocked linker is highly similar to the crystallized form (Figure 5C cf. 5D) showing that the junctions between subdomains are robust. Only rarely is a bend visible within the undocked linker, occurring between subdomains 2 and 3 when the linker lies to the left side of the ring (Figure 5B, arrow). In summary these undocked molecules show that the linker is a stable domain when detached from the ring, indicating that hinging within the linker is actively promoted by interactions with the AAA+ modules.

### Models of Complete Dynein-c and Tail Flexibility

Remodeling of the linker results in large-scale displacement of the tail relative to the microtubule-binding domain at the stalk's tip. To understand these relative movements, we built complete models of dynein-c in the primed and unprimed states using our cryo-EM maps, crystal structures of the stalk (Carter et al., 2008; Kon et al., 2012), and a reconstruction of the tail (Figure S1I), for which no crystallographic structure has been published. The resulting models satisfy independent constraints from different experimental methods (see Experimental Procedures for details of model building; Figure S4). The primed and unprimed models are differentiated by a 65°–70° rotation of the distal linker and tail, relative to the linker's base. The motion of these elements is approximately in the plane of the ring. The primed linker emerges from the ring principally over AAA3, proximal to AAA4's H3-β4 insert (Kon et al., 2012; Schmidt et al., 2012). During the powerstroke transition the molecule undergoes a net end-to-end contraction of ∼4 nm.

**Figure 6:**
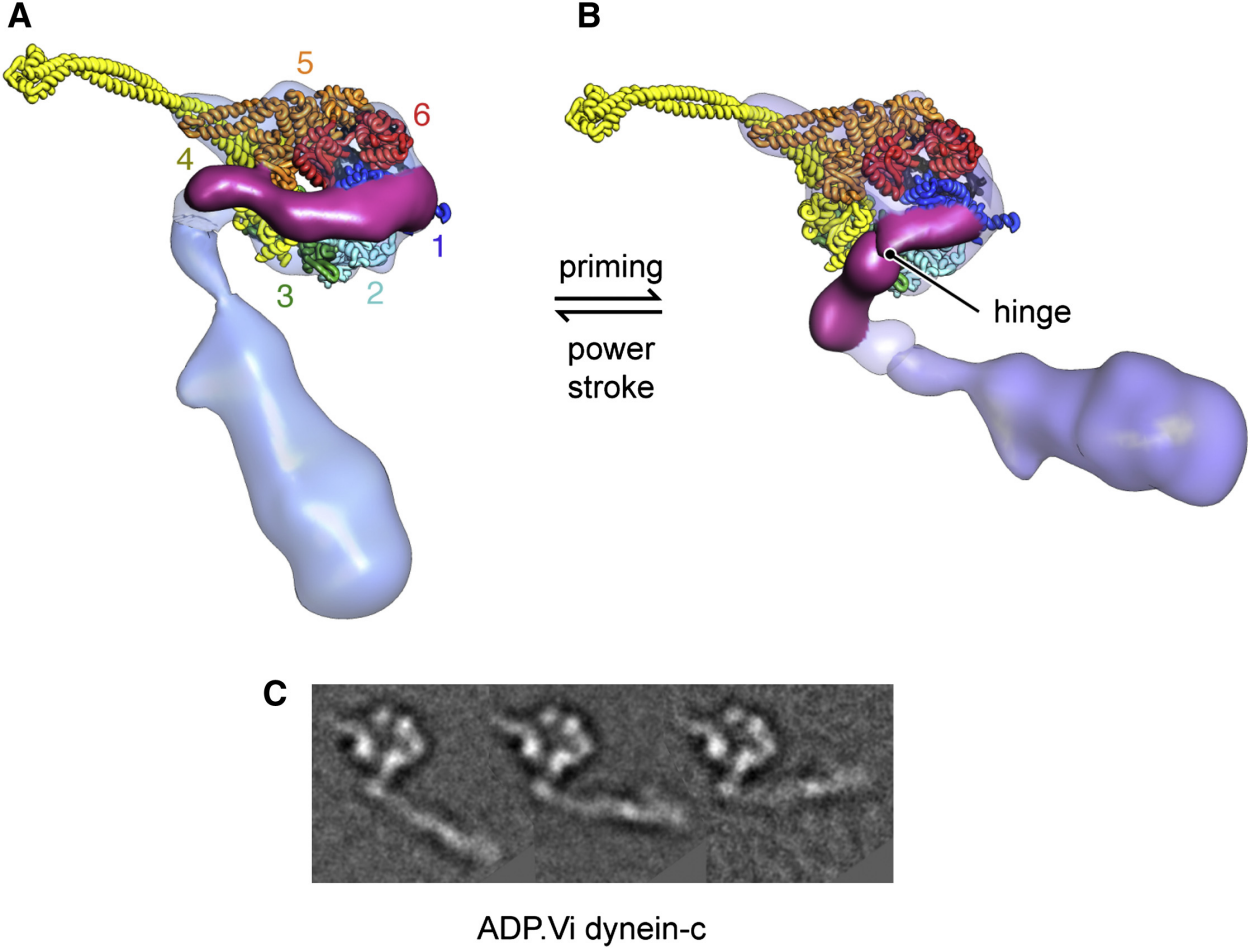
Complete Models of Dynein-c and Tail Flexibility (A and B) Complete models of dynein-c. A reconstruction of the tail domain is docked onto the cryo-EM head map in the absence of nucleotide (A) and in the ADP.Vi state (B). For details of model building, see Experimental Procedures and Figure S4. Chain B of the *D. discoideum* dynein crystal structure PDB 3VKG (Kon et al., 2012) is docked into the head maps, and the microtubule-binding domain is from PDB 3ERR (Carter et al., 2008). The linker domain is colored magenta. (C) Cryo-EM class averages showing the tail domain of ADP.Vi dynein-c at a range of angles relative to the head. The head orientation is the same as in (B). See also Movie S3.

Previous negative-stain EM studies have suggested that flexibility within the neck subdomain of the tail could cause dynein-c's powerstroke to cover a range of sizes (Burgess et al., 2003, 2004a). However, to what extent the apparent tail flexibility arose from substrate adsorption and negative staining was unclear. We therefore analyzed the tail using our unstained cryo-EM data, applying image classification to group molecular images based on tail features. The resulting class averages show that the tail adopts a range of angles relative to the head (Figure 6C) with a SD of ±10° (see also Movie S3). This demonstrates that tail flexibility is a bona fide feature of dynein's molecular structure, indicating that the angle of the tail can vary in response to thermal fluctuations or external load (see below).

### Docking Dynein-c into Cryo-Electron Tomograms of the Axoneme

**Figure 7:**
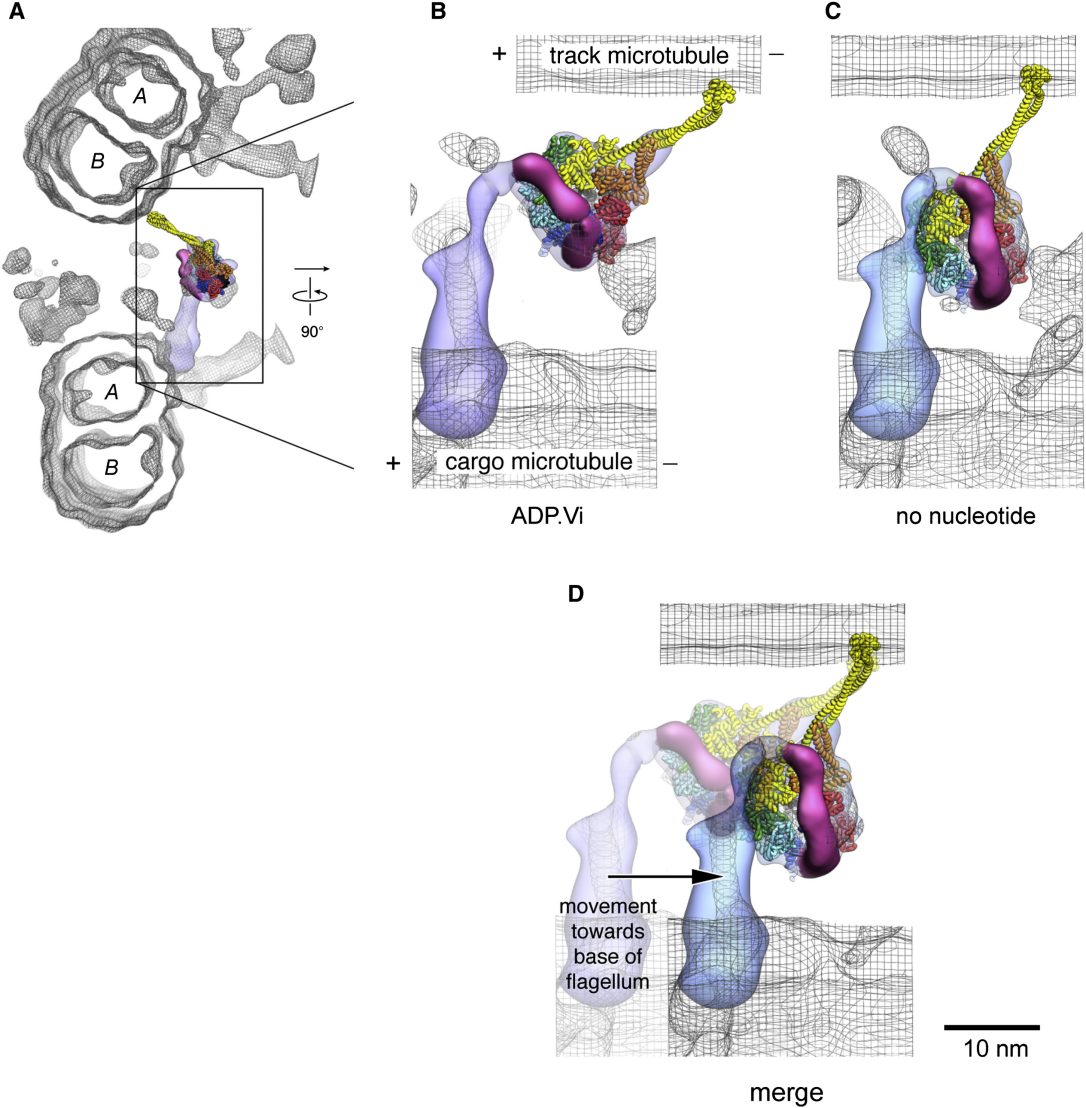
Dynein-c Structure in the Axoneme (A and B) Views of the primed dynein-c model (Figure 6B) docked into an averaged tomogram of ADP.Vi-dynein-c in the axoneme (Movassagh et al., 2010) (wire mesh). The quality of the fit is best seen in Figure S5A. The linker domain is colored in magenta. Plus (+) and minus (−) signs show microtubule polarity; plus ends are at the tip of the axoneme. The “*A*” and “*B*” microtubules in each doublet are labeled. (C) View of the unprimed dynein-c model docked into an averaged tomogram of dynein-c in the axoneme without nucleotide (wire mesh) (Bui et al., 2009). (D) Overlay, superposing the tips of the stalks of the two dynein-c models, with nondynein-c densities removed for clarity. The arrow depicts the inferred displacement of the “cargo” microtubule doublet past the adjacent “track” doublet. The view in (B)–(D) is from the outside of the axoneme, looking in. The view in (A) is from the base of the axoneme, looking toward the tip.

To elucidate how the structural changes in dynein-c are harnessed to produce sliding between microtubules in the flagellum, we docked our complete models into tomograms of the *C. reinhardtii* axoneme in ADP.Vi and no-nucleotide conditions (Bui et al., 2009; Movassagh et al., 2010). The dynein-c density in the tomograms matches the overall shape of the head and tail, though the stalk and linker are not resolved in the tomographic maps (Figures 7 and S5). Hence, the docking provides new insights into how these elements produce translocation of one “cargo” microtubule doublet toward the minus end of an adjacent “track” doublet. Such sliding movements are resisted by other axonemal components in situ, converting the motions into bending deformations.

The primed dynein-c model fits well into the head and tail density of the ADP.Vi tomogram without further modification (Figure S5A) and nicely places the tip of the stalk close to the surface of the adjacent track microtubule (Figures 7A and 7B). The model's tail is almost perpendicular to the cargo microtubule on which it is anchored. The linker lies on the outer face of the AAA+ ring (i.e., facing the flagellar membrane). From the head the stalk is angled toward the minus end of the track microtubule, consistent with observations from outer-arm and cytoplasmic dyneins (Carter et al., 2008; Ueno et al., 2008). This model is thus probably close to dynein structure at the start of its powerstroke.

Regarding the end of the powerstroke, when the unprimed dynein-c model is fitted into the nucleotide-free tomogram based on the tail density (which remains perpendicular to the cargo microtubule), there is an imperfect fit of the head domain (Figure S5B). This indicates that the conformation of the head and tail domains in the axoneme is modified from the mean orientation in isolated dynein-c. A good fit with the tomogram can be made by introducing a bend of 35° into the flexible neck subdomain of the tail (Figure S5B). The resulting head-tail disposition lies within the range seen in isolated dynein-c molecules (Burgess et al., 2004a). In the refined model (Figure 7C), the stalk remains inclined toward the minus end of the track microtubule but less steeply than in the primed structure. The strut lies on the correct side of the stalk to support it in the presence of a load that opposes the powerstroke, suggesting that it can indeed act as a strut. The powerstroke of dynein-c shown in Figure 7D produces an overall displacement of ∼12 nm.

## Discussion

Using cryo-EM, protein engineering, and domain fitting, we have obtained results that provide several insights into the mechanism of dynein motility. Moreover, we demonstrate that dynein's structure and flexibility can be analyzed in near-native conditions by cryo-EM and image processing. This is significant because cryo-EM provides an ideal complement to X-ray crystallography, the latter providing high-resolution structural information but typically of only one “fleximer” (Burgess et al., 2004a) that makes good crystal contacts. Because our results indicate that dynein's flexibility and dynamics have a critical bearing on the motile mechanism, cryo-EM is likely to play an important role in elucidating the molecular basis for dynein's wealth of biological functions.

### Axonemal Dynein Structure

In our map of native dynein-c prepared in the absence of nucleotide, the nucleotide-binding pocket at AAA1 is open, the linker interacts with AAA5, and there is an unexpected contact between the neck subdomain of the tail and AAA4. The effect of ADP.Vi binding is dramatic, causing all these features to change. The role of the interaction between the neck and AAA4 is not yet clear, but it may help to stabilize the linker position in the no-nucleotide state. Because the neck is not part of the minimal dynein motor domain, this neck-ring interaction does not appear essential for dynein movement, although it could regulate motility. For example in the case of cytoplasmic dynein, the native protein generates ∼40% more force than the motor domain lacking the tail (Gennerich et al., 2007).

Our structures of purified dynein-c generally show excellent agreement with the dynein-c density in cryo-electron tomograms of the *C. reinhardtii* axoneme. This docking extends previous analyses that involved fitting idealized ring shapes into the tomograms prior to the availability of higher-resolution structures (Bui et al., 2008; Movassagh et al., 2010). Notably, a bend of 35° within the neck of our structure was required to fit the tomogram in the nucleotide-free state. This is in agreement with the findings of Movassagh and coworkers, who reported a change in neck shape of dynein-c between ADP.Vi and nucleotide-free tomograms (Movassagh et al., 2010). Furthermore, our image classification of purified dynein-c shows that the neck is a natural site of flexibility within the molecule, reinforcing earlier results from negative-stain EM (Burgess et al., 2003, 2004a). Interestingly, a report suggests that the tail flexibility in outer-arm dynein is regulated by calcium-dependent interactions with the LC4 light chain (Sakato et al., 2007). Together, these results indicate that the neck is a flexible structure that can be strained by forces in the axoneme and might also be acted on by dynein cofactors.

### Mechanism of Linker Movement and Undocking

Our cryo-EM structures of axonemal and cytoplasmic dynein provide evidence that ATP binding at AAA1 causes hinging within the linker. This hinge site is near a cleft previously identified between linker subdomains (Kon et al., 2012; Schmidt et al., 2012). The finding that the distal linker moves with respect to the base provides an explanation for earlier in vitro motility studies, in which the microtubule-gliding velocity dropped close to zero when dynein was tethered via sites that can now be mapped to the linker's base (Shima et al., 2006).

Undocked from the AAA+ ring, the linker domain appears as a mechanically stable entity. This implies that hinging within the linker is strongly promoted by interaction with the AAA+ modules. Supporting this, the bend we find within the primed linker is sharper and larger than that predicted from normal mode analysis of the unprimed structure (Zheng, 2012). Crystallographic analysis of the motor domain in the ADP state has revealed that two β hairpins within AAA2 contact the linker on either side of the cleft site, making these motifs strong candidates to mediate hinging (Kon et al., 2012). In ADP.Vi dynein-c the linker domain does not exclusively adopt a primed position, perhaps because the linker exists in a dynamic equilibrium in this state as previously considered (Roberts et al., 2009). This could, in principle, enable dynein to sample a range of binding sites on the microtubule during the weak binding phase of its mechanochemical cycle (see below).

The linker movement suggested by our data implies interesting similarities and differences with the mechanical elements in the other cytoskeletal motor families (Vale and Milligan, 2000). In the case of kinesin-1, the neck linker is a small mobile polypeptide that docks onto the catalytic domain upon ATP binding. In contrast the lever domain of myosin appears closer in mechanical properties to dynein's linker but rotates as a rigid body to amplify movement rather than being actively reshaped. Thus, the mechanism of linker motion in dynein appears conceptually closer to the actions of other AAA+ machines, which also use ATP to remodel mechanically stable substrates. Interestingly, however, the specifics of the remodeling reaction can vary widely within the superfamily. For instance some AAA+ proteins such as unfoldases use loops in the lumen of their ring to perform vectorial work on their substrates (Martin et al., 2008), whereas others like PspF (Rappas et al., 2005) and dynein (Kon et al., 2012) grip their targets using inserts on the face of the AAA+ ring.

In this study we discovered conditions that cause the linker to frequently become detached from the AAA+ ring. This ADP-induced effect is seen in a construct bearing a truncated C-terminal domain, making it similar in length to naturally shorter fungal dyneins. Forces associated with surface adsorption of the protein during negative staining may also have been involved in eliciting linker undocking. In this regard it is interesting to consider whether the linker can undock from the ring under physiological conditions. For example might the forces imparted by macromolecular cargo, a partnering motor, or the actively beating flagellum cause linker undocking? One clue comes from single-molecule experiments that indicate that the distance between heads in cytoplasmic dynein dimers is able to exceed the maximum separation expected from molecules with docked linkers (DeWitt et al., 2012; Qiu et al., 2012).

### Implications for Microtubule-Based Movement

**Figure 8:**
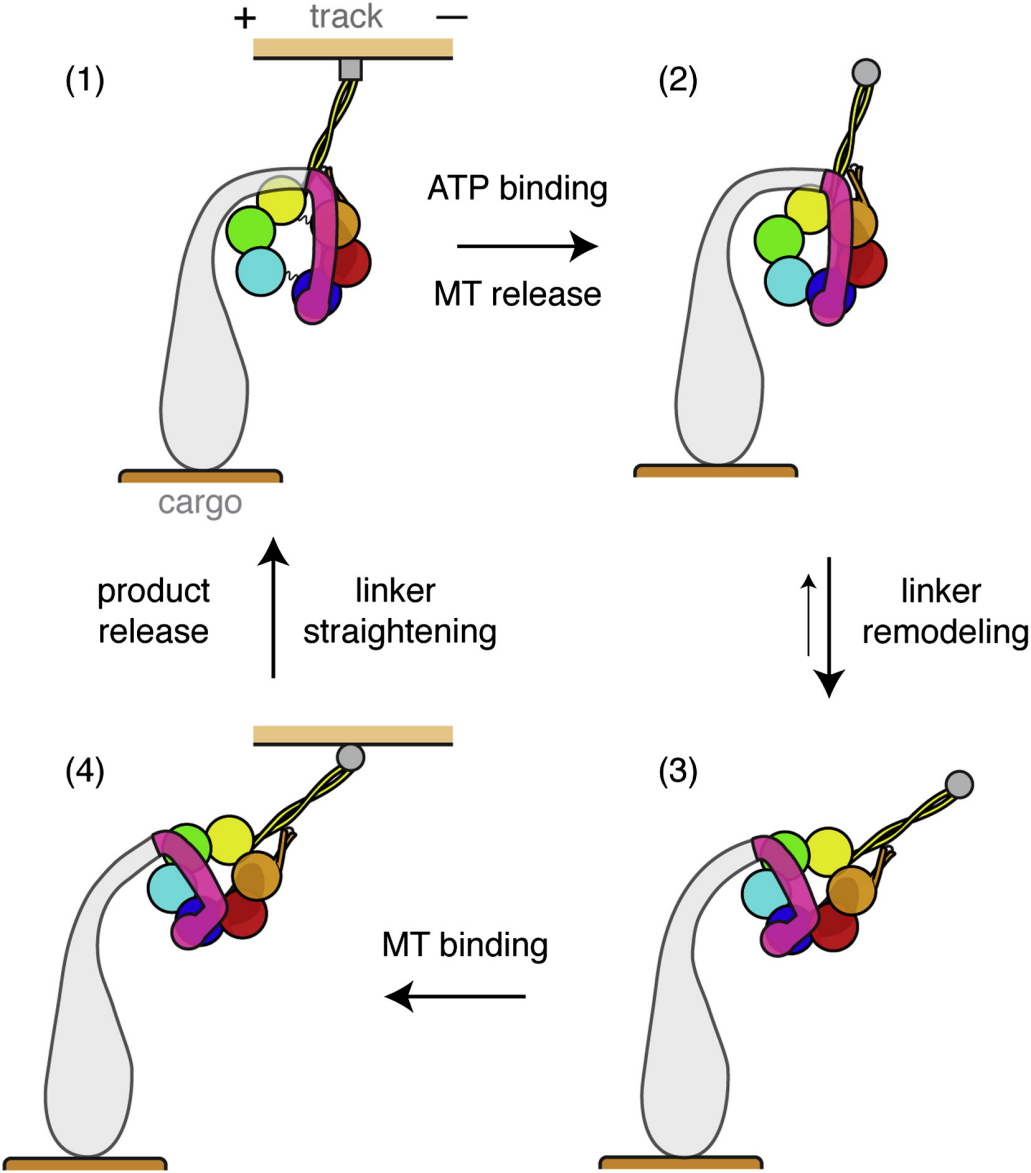
Model Cycle for How Structural Changes in Dynein Produce Motility See text for details and discussion of possible intermediate states. MT, microtubule. Plus (+) and minus (−) signs indicate microtubule polarity.

We bring our results and previous studies together into a refined model for the mechanochemical cycle of dynein-c (Figure 8). Our analysis of cytoplasmic and axonemal dyneins indicates that the core features of the mechanism are common to both.

(1) With its main ATPase site (AAA1) empty, dynein is bound tightly to the microtubule track. The linker and neck are latched onto AAA5 and AAA4 in the ring, respectively.
(2) The binding of ATP between AAA1 and AAA2 causes the large gap between these modules to close. This event begins a complex set of domain motions within the ring. One clear change is a rearrangement involving AAA4 and AAA5, which may dislodge the linker and neck. A second likely consequence is to alter interactions between the stalk (embedded in AAA4) relative to the strut (embedded in AAA5), leading to a structural change within the stalk coiled coil and microtubule release (Gibbons et al., 2005; Kon et al., 2012).
(3) A slower, ATP-induced step is the priming of the linker (Imamula et al., 2007), which shifts to lie over AAA3. Our data indicate that this occurs via hinging between the distal linker and its base. With the tail anchored (e.g., to a cargo microtubule or a viscoelastic cargo), the hinging within the linker lengthens the reach of the stalk along the microtubule. Conformational fluctuations in the molecule, particularly at the linker and neck, would vary the amount of movement and might contribute to the variety of observed step sizes (Sakakibara et al., 1999).
(4) In this weak-binding conformation, the stalk attaches to a new site on the microtubule. The orientation of our whole molecule model into the flagellar tomogram shows that binding sites in which the stalk is angled toward the microtubule minus end are likely to be favored. Strong binding of the stalk to the microtubule allosterically activates the release of hydrolysis products from AAA1 (Kon et al., 2009) and causes the linker to move toward the stalk, presumably driven by a straightening of its structure (e.g., a reversal of the remodeling process).

It remains to be discovered if the linker movement occurs in stages, associated with the sequential release of P_i_ and ADP, as is the case with the lever in some myosins. Conversely, the majority of movement may occur after P_i_ release, and a small shift of the linker, from AAA4 to AAA5, may be involved in wedging open the ring and promoting the release of ADP in some dyneins (Schmidt et al., 2012). The net result of the linker straightening is that the molecule contracts, pulling the cargo both along the track and also toward it. An important question for future investigation is how cytoplasmic dynein, with its two identical motor domains, can harness this linker action to produce a processive stepping machine.

## Experimental Procedures

### Specimen Preparation

Dynein-c was purified from *C. reinhardtii* flagella as described by Sakakibara et al. (1999) and diluted into Buffer A (10 mM MOPS, 3 mM MgCl_2_, 1 mM EGTA [pH 7.5]) to a final concentration of ∼75–190 μg/ml protein. Residual KCl from the elution buffer was 100–250 mM. To produce ADP.Vi-dynein-c, 100 μM ATP and 50 μM Vi were added, and the mixture was incubated on ice for 30 min.

*D. discoideum* cytoplasmic dynein constructs with N-terminal purification tags (His6 and FLAG) were prepared as described by Kon et al. (2004). Amino acid numbering within the heavy chain is based on DDB0185096 in dictyBase (Kon et al., 2012). For the unprimed conformation the 380 kDa motor domain (V1388–I4730) was diluted into Buffer B (10 mM PIPES-KOH, 50 mM potassium acetate, 4 mM MgSO_4_, 1 mM EGTA, 1% glycerol [pH 7.0]) with 1 mM ADP. For the primed conformation the motor domain bearing a E2027Q substitution was diluted into Buffer B supplemented with 1 mM ATP.

In some preparations, F-actin or taxol-stabilized microtubules were added to improve the spread of the specimen on the EM grids. For dynein-c in the absence of nucleotide, samples were treated with apyrase (0.14 U/ml) for 15 min to remove ATP and ADP from the actin preparation.

### Cryo-EM

Specimen (3 μl) was applied to glow discharged, holey carbon film (Quantifoil Micro Tools GmbH, Jena, Germany), or lacey carbon film (Agar Scientific) on copper grids. After blotting from both sides with filter paper, grids were flash frozen in liquid ethane and stored under liquid N_2_. Grids were transferred using a Gatan 626 holder into a Tecnai F20 microscope with a field emission gun operating at 200kV. Low-dose micrographs were collected at a range of defocus values (∼0.5–5 μm) using a Gatan US4000SP CCD camera and a calibrated magnification of 69,444× (giving a final object sampling of 2.16 Å/pixel). For absolute hand determination, tilt pair micrographs (at 0° and +15°) of vitrified dynein-c were collected. Tilt pair micrographs of negatively stained GroEL (a kind gift from Dr Peiyi Wang) served as control images of an object with known handedness. For details of negative-stain EM of the C-terminally truncated cytoplasmic dynein construct, see Supplemental Experimental Procedures.

### Image Preprocessing

Micrograph defocus was determined using the program CTFFIND3 (Mindell and Grigorieff, 2003). Particle coordinates, centered on dynein's head domain, were picked manually using the EMAN program BOXER (Ludtke et al., 1999) or the SPIDER program WEB (Frank et al., 1996). Using SPIDER, particles were excised into windows, phases were corrected for the effects of the contrast transfer function, and particles were band-pass filtered between 6.6 and 300 Å. The number of particles in each data set was 24,656 (dynein-c, no nucleotide), 30,597 (dynein-c, ADP.Vi); 35,571 (cytoplasmic motor, ADP), and 25,715 (cytoplasmic motor [E2027Q], ATP).

### Image Processing and Structure Refinement

We obtained a starting model de novo using the nucleotide-free dynein-c data set. Particles were treated with a soft-edged circular mask outside the head domain, aligned according to features within the head by reference-free methods then, using IMAGIC software (van Heel et al., 1996), classified into 1,000 classes by multivariate statistical analysis. The relative angular orientations of eight selected classes were determined computationally by angular reconstitution in IMAGIC. Chosen classes included views of the head previously characterized in negative stain and those in which the emergence of the tail from the head was visible, the position of which allowed visual validation of the angular assignments. A total of 21 iterative rounds of refinement was carried out by projection matching in SPIDER, first using the 1,000 class averages and angular intervals of 20° between projections and finally using individual particles and intervals of 5°. Due to the presence of preferred orientations (Figure S1), the maximum number of particles contributing to each angular view was restricted to the total number of particles divided by the total number of views. During refinement the mean cross-correlation scores (between individual particles and reprojections) increased, and the number of images changing their Euler angle assignments decreased, until negligible further changes were seen, and the structure was judged to have converged. To gain qualitative insight into the location of variable regions in the map, variance images were calculated for each angular view and back projected. The absolute hand of the structure was determined using tilted images according to the method of Rosenthal and Henderson (2003), implemented in SPIDER software.

For ADP.Vi-dynein-c we used as a starting model the no-nucleotide dynein-c density map. A total of 25 iterations of projection matching was performed in SPIDER using 5° intervals between projections and individual particles. By the tenth iteration of refinement, density for part of the unprimed linker disappeared, and no corresponding reappearance in a new position was found, prompting us to intervene in the refinement by reducing the angular resolution to 10° (iterations 26–31) before restoring it to 5° (iterations 32–38). This did not cause the missing part of the linker to reappear. We also tested alternative refinements at 20° intervals and using earlier no-nucleotide dynein-c models in which the linker was less well defined, all of which converged to a highly similar final structure (shown). For details of cytoplasmic dynein refinement and reconstruction of dynein-c's tail domain, see Supplemental Experimental Procedures.

### Model Building

Construction of complete dynein-c models began with docking the tail reconstruction onto the cryo-EM head map in the nucleotide-free state, based on the cryo-EM-derived variance map. The tail position was then compared to that in previously characterized negative-stain views of dynein-c, after the orientation of the cryo-EM head map corresponding to each view was determined by cross-correlation. The agreement in the tail position between the two independent methods was striking (Figure S4). To model the stalk, the *D. discoideum* cytoplasmic dynein crystal structures (Kon et al., 2012) were docked into the cryo-EM map in a semiautomated fashion using UCSF Chimera (Pettersen et al., 2004). Chain B of PDB 3VKG showed a stalk angle in excellent correspondence with earlier class averages of dynein-c (Figure S4). Because the microtubule-binding domain is deleted in this structure, a separate structure of the microtubule-binding domain and distal coiled coil (PDB 3ERR) (Carter et al., 2008) was attached in register. The resolution of our cryo-EM maps did not permit readjustments of the AAA+ module positions from the crystal structure, so their location in the model is approximate. To generate the primed model, the distal linker and tail were first segmented from the nucleotide-free structure. These elements were then rotated together as a rigid body relative to the base of the linker in the ADP.Vi structure, based on the cryo-EM-derived variance map, earlier negative-stain views, and cryo-EM class averages of ADP.Vi dynein-c in which the tail was directly visible (Figure 6C). The final linker and tail position satisfies all these constraints. A small readjustment in the stalk angle was made to account for the curvature of dynein-c's stalk in the ADP.Vi state (Burgess et al., 2003).

### Structure Visualization

Final maps were filtered to their resolution (according to the 0.5 Fourier shell correlation criterion) and displayed at thresholds determined by comparisons to crystal structures. The linker domain was segmented using the Chimera plug-in Segger. UCSF Chimera (Pettersen et al., 2004) was used for rigid body fitting of crystal structures (Kon et al., 2012; Schmidt et al., 2012) into the cryo-EM maps. Figures were prepared using Chimera and PyMOL software (Schrödinger, 2010).

## Accession Numbers

The cryo-EM maps of dynein-c in the absence of nucleotide and the ADP.Vi state have been deposited to the EMDataBank with accession codes of EMD-2155 and EMDB-2156, respectively.

## Supplemental Information

Document S1. Figures S1–S5 and Supplemental Experimental Procedures

Movie S1. Sequence of Class Averages Showing the Undocked Linker in the Motor-yC Construct, Related to Figure 5

Movie S2. Sequence of Linker Class Averages, following Alignment Based upon Undocked Linker Features, Related to Figure 5

Movie S3. Sequence of Cryo-EM Class Averages Showing Flexibility of the Tail Domain in Dynein-c, Related to Figure 6
