# Supplementary material for: ATP-Driven Remodeling of the Linker Domain in the Dynein Motor
Source: Structure. 2012 Oct 10;20(10):1670–80. doi: 10.1016/j.str.2012.07.003 (PMC3469822; doi:10.1016/j.str.2012.07.003)
Supplement: Document S1. Figures S1–S5 and Supplemental Experimental Procedures [file mmc1.pdf]

Structure, Volume *20*

Supplemental Information

ATP-Driven Remodeling of the Linker Domain in the Dynein Motor

Anthony J. Roberts, Bara Malkova, Matt L. Walker, Hitoshi Sakakibara, Naoki Numata, Takahide Kon, Reiko Ohkura, Thomas A. Edwards, Peter J. Knight, Kazuo Sutoh, Kazuhiro Oiwa, and Stan A. Burgess

<u>Inventory of Supplemental Information</u>

Supplemental Data, comprising:

Figure S1. Related to Experimental Procedures

Figure S2. Related to Figure 1

Figure S3. Related to Figure 5

Figure S4. Related to Figure 6

Figure S5. Related to Figure 7

Movies S1 and S2. Related to Figure 5

Movie S3. Related to Figure 6

Supplemental Experimental Procedures

Supplemental References

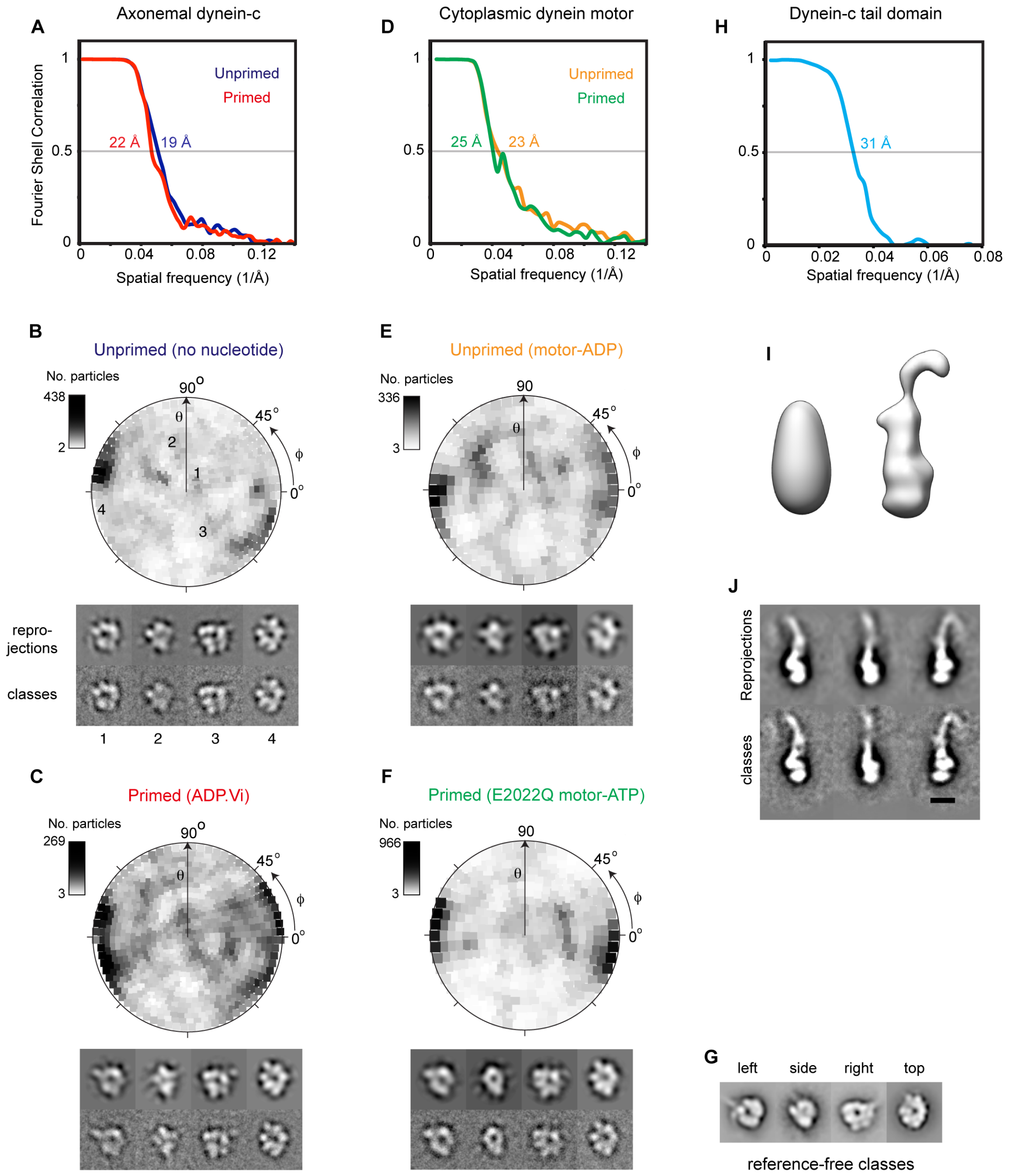

Figure S1. 3D Refinement, Resolution and View Coverage, Related to Experimental Procedures
Fourier Shell Correlation (FSC) curves for axonemal (**A**) and cytoplasmic (**D**) dynein cryo-EM reconstructions and a negative-stain reconstruction of the tail domain (**H**). Resolutions according to the 0.5 FSC criterion are indicated. (**B, C, E, F**) Polar plots of the angular distribution of views in each cryo-EM dataset, showing preferred orientations (darker regions) but full coverage of Euler space (φ = rotation about z, θ = rotation about y). Consistency between reprojections of each 3D map and the corresponding input class averages is shown below. The four views in B (1-4) are also mapped on the polar plot. (**G**) Views of dynein-c (left, side and right views) and the cytoplasmic dynein motor domain (top view) from previous negative stain studies (Burgess et al., 2003; Burgess et al., 2004; Roberts et al., 2009). (**I**) Tail domain starting model (left panel) and final map after refinement (right panel). (**J**) Consistency between reprojections of the tail 3D map and the corresponding input class averages. Scale bar is 10 nm.

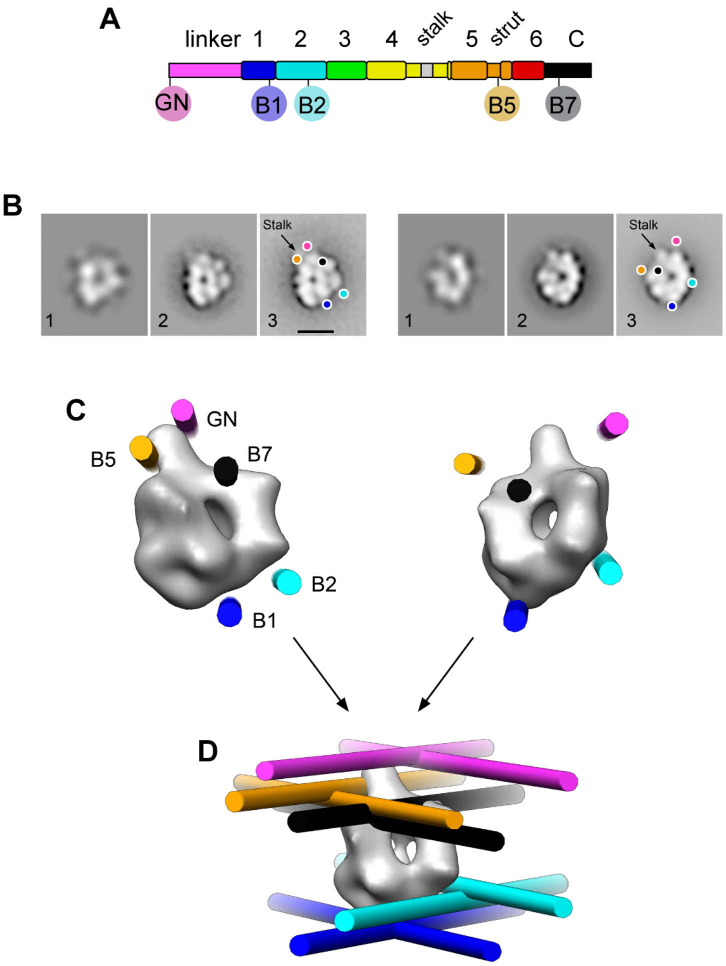

Figure S2. Integration of Cryo-EM Maps with Earlier Mapping Data, Related to Figure 1

(**A**) Sequence diagram of the 380 kDa cytoplasmic dynein motor domain from *D. discoideum*. Insertion sites of a GFP tag at the N terminus (GN) and four BFP tags previously engineered within the head (B1, B2, B5, and B7) are indicated (Roberts et al., 2009).

(**B**) To map the tag positions, reprojections of the cytoplasmic dynein motor domain cryo-EM map were matched to right and top views of the motor from negative stain EM (panel 2) by cross correlation. Panel 1 shows the reprojections with the highest cross correlation score. Panel 3 shows the mean positions of the tags previously mapped in each 2D view.

(**C**) The tag positions were used to insert cylinders along the corresponding axes in the 3D volumes. The cylinder radius is 1 nm.

(**D**) When the mapping data from both views are combined (bringing the maps in C into mutual register), the pairs of cylinders for each tag intersect. If the assigned axis of rotation between the top and right views were erroneous, the cylinders would not intersect. The points of intersection give the approximate 3D position of each tag according to this analysis. The position of the GN tag in the primed motor was similarly determined using the primed cryo-EM map and 2D views of the motor in the ADP.Vi state from negative stain (Roberts et al., 2009).

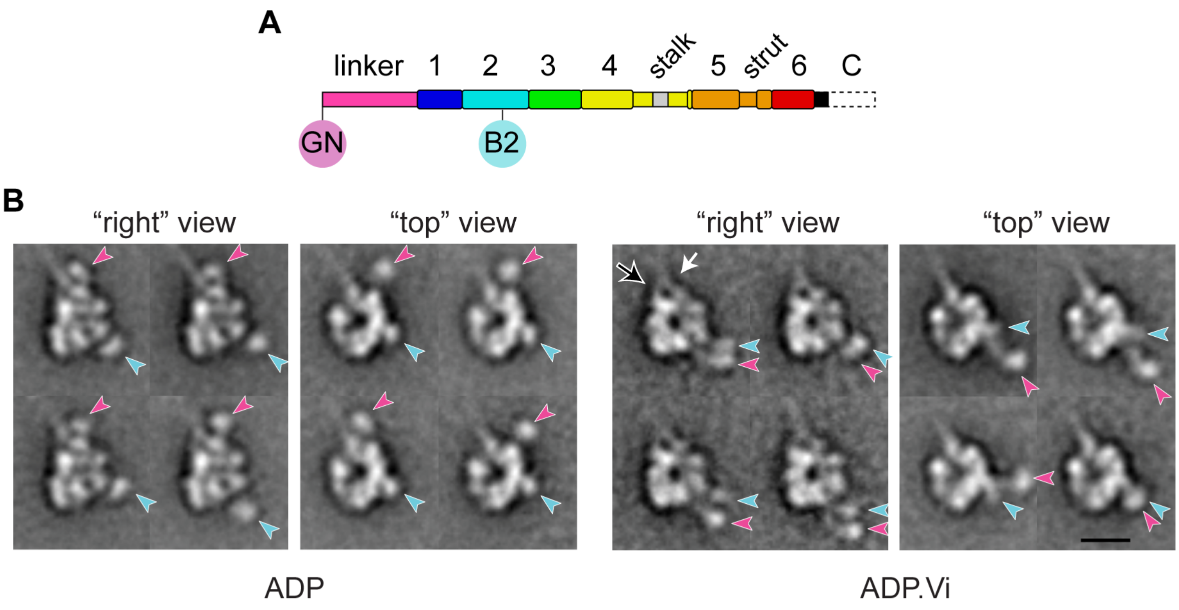

Figure S3. Linker Movement in a C-terminally Truncated Motor, Related to Figure 5

(**A**) Diagram of the cytoplasmic dynein construct from *D. discoideum* with a C-terminal truncation (dashed outline, corresponding to amino acids 4448-4730). We refer to this construct as 'motor-yC' as its C-terminus is approximately equivalent to the naturally shorter yeast cytoplasmic dynein. Insertion sites of GFP at the N-terminus (GN) and BFP within AAA2 (B2, after S2476) are depicted.

(**B**) Negatively stained motor-yC molecules in the presence of ADP or ATP plus vanadate (Vi, to create ADP.Vi-dynein) are seen in both right and top views, as previously described for the intact motor domain. In ADP, most molecules (~66%) show the undocked appearance, in which the linker domain is separated from the ring (Figure 5, main text). In the remaining ADP molecules, the GN tag (magenta arrowheads) is found in the unprimed position near the base of the stalk. With ADP.Vi bound, linker undocking is not seen, and the GN tag lies near the B2 tag (cyan arrowheads) inserted in AAA2. These linker positions are consistent with those reported for the intact motor domain (Roberts et al., 2009). In ADP.Vi right views, the stalk coiled coil (white arrow) and strut (black arrow) are seen. Scale bar is 10 nm.

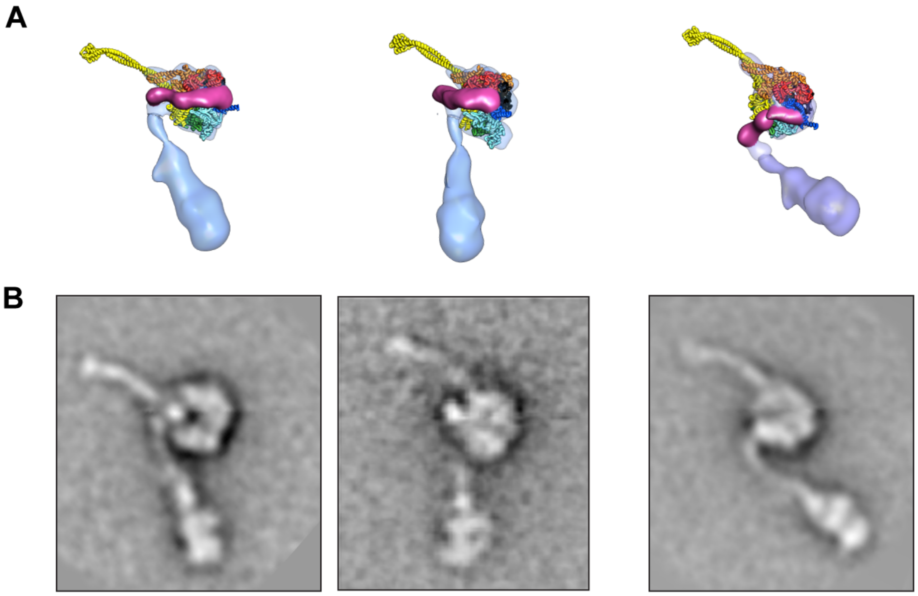

Figure S4. Comparison of Dynein-c Models to Earlier Negative Stain Views, Related to Figure 6

Panels show consistency between (**A**) models of dynein-c in no nucleotide and ADP.Vi states, constructed as described in Experimental Procedures, and (**B**) equivalent views of dynein-c by negative stain EM (Burgess et al., 2003; Burgess et al., 2004).

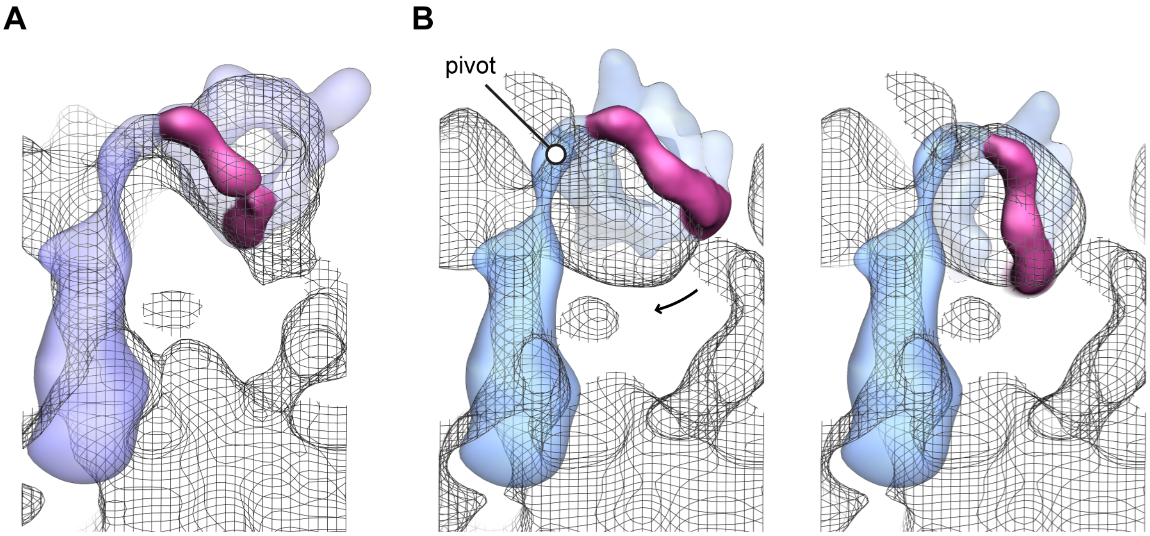

Figure S5. Further views of Dynein-c Docked into Axonemal Tomograms, Related to Figure 7

(**A**) View of the primed dynein-c model docked into an averaged tomogram of ADP.Vi-dynein-c in the axoneme (Movassagh et al., 2010) (wiremesh).

(**B**) Fitting of the unprimed dynein-c model into the tomogram of nucleotide-free dynein-c in the axoneme (Bui et al., 2009) requires a 35° rotation about the indicated pivot within the flexible neck subdomain of the tail. See Discussion for details.

SUPPLEMENTAL MOVIE LEGENDS

Movie S1, Related to Figure 5.
Sequence of class averages showing the undocked linker in the motor-yC construct. The linker, with N-terminal GFP at its distal end, adopts a variety of positions relative to the ring, flexing predominantly within the thin connection between linker and ring (seen when the linker lies to the right of the ring). The connection between N-terminal GFP and the linker is also flexible, while the linker domain itself appears rather stiff (see Supplementary Movie 2). Scale bar is 10 nm.

Movie S2, Related to Figure 5.
Sequence of linker class averages, following alignment based upon undocked linker features. Comparison between the class averages indicates that the cleft between linker subdomains (arrowhead) is robust when the linker is undocked. The classes differ mainly in the position of GFP, which is attached to the linker N-terminus via a flexible spacer sequence. Scale bar is 10 nm.

Movie S3, Related to Figure 6.
Sequence of cryo-EM class averages of ADP.Vi-dynein-c with the linker in the primed position, showing the tail domain at a range of angles relative to the head. Flexibility occurs within the neck region, where the tail joins the linker. Scale bar is 10 nm.

SUPPLEMENTAL EXPERIMENTAL PROCEDURES

Cytoplasmic Dynein Refinement

Reference-free cryo-EM class averages of the cytoplasmic dynein motor, generated using the EMAN program *refine2d.py*, showed strong similarity with reprojections of the dynein-c cryo-EM maps (data not shown). Cryo-EM maps of dynein-c in the no nucleotide and ADP.Vi states were therefore used as starting models to refine the cytoplasmic dynein motor-ADP and motor(E2027Q)-ATP datasets respectively. The starting models were low-pass filtered to a resolution of 50 Å to prevent model bias at higher resolution. Twenty iterations of projection matching were carried out using EMAN software and angular spacing of 7° between projections. At each iteration, particles assigned to a given orientation were subjected to six rounds of reference-free alignment.

Tail Reconstruction

A 3D reconstruction of the tail domain was obtained using images of negatively stained dynein-c molecules (Burgess et al., 2003; Burgess et al., 2004). To produce images in which the prominent feature was the tail domain, a normalized average of the head domain was subtracted from each particle image. For angular assignment, an egg-shaped volume (Figure S1I) was used as a starting model for projection matching in EMAN software. Reference views were generated around the long axis of the model and tilted up to 7º from the equator. In each iteration, particles assigned to a given orientation were subjected to six rounds of reference-free alignment. During refinement, density for the neck appeared, despite not being present in the starting model (Figure S1I). The absolute hand of the tail reconstruction is unknown and does not impact on the results presented.

Negative Stain Electron Microscopy

The C-terminally truncated *D. discoideum* cytoplasmic dynein construct was purified, negatively stained and imaged by electron microscopy using described methods (Numata et al., 2011; Roberts and Burgess, 2009). A total of 4,984 particles in the presence of ATP and Vi were aligned and classified. A total of 42,841 particles in the presence of ADP were aligned, classified and separated into undocked (13,949), right (5,950) and top (1,289) views. The remaining particles were either partially stained or of as yet uncharacterized views. From the undocked molecules, 9,850 particles were selected with the undocked linker visible to the right of the ring and 1,263 particles with the undocked linker to the left of the ring; the remaining particles had the linker only partially stained.

SUPPLEMENTAL REFERENCES

Bui, K.H., Sakakibara, H., Movassagh, T., Oiwa, K., and Ishikawa, T. (2009). Asymmetry of inner dynein arms and inter-doublet links in Chlamydomonas flagella. J. Cell Biol. *186*, 437-446.

Burgess, S.A., Walker, M.L., Sakakibara, H., Knight, P.J., and Oiwa, K. (2003). Dynein structure and power stroke. Nature *421*, 715-718.

Burgess, S.A., Walker, M.L., Sakakibara, H., Oiwa, K., and Knight, P.J. (2004). The structure of dynein-c by negative stain electron microscopy. J. Struct. Biol. *146*, 205-216.

Movassagh, T., Bui, K.H., Sakakibara, H., Oiwa, K., and Ishikawa, T. (2010). Nucleotide-induced global conformational changes of flagellar dynein arms revealed by in situ analysis. Nat. Struct. Mol. Biol. *17*, 761–767.

Numata, N., Shima, T., Ohkura, R., Kon, T., and Sutoh, K. (2011). C-Sequence of the Dictyostelium cytoplasmic dynein participates in processivity modulation. FEBS Letters *585*, 1185-1190.

Roberts, A.J., Numata, N., Walker, M.L., Kato, Y.S., Malkova, B., Kon, T., Ohkura, R., Arisaka, F., Knight, P.J., Sutoh, K., and Burgess, S.A. (2009). AAA+ ring and linker swing mechanism in the dynein motor. Cell *136*, 485-495.

Roberts, A.J., and Burgess, S.A. (2009). Electron microscopic imaging and analysis of isolated dynein particles. Methods Cell Biol. *91*, 41-61.
