# Supplementary figures and images for: ATP-Driven Remodeling of the Linker Domain in the Dynein Motor
Source: Structure. 2012 Oct 10;20(10):1670–80. doi: 10.1016/j.str.2012.07.003 (PMC3469822; doi:10.1016/j.str.2012.07.003)

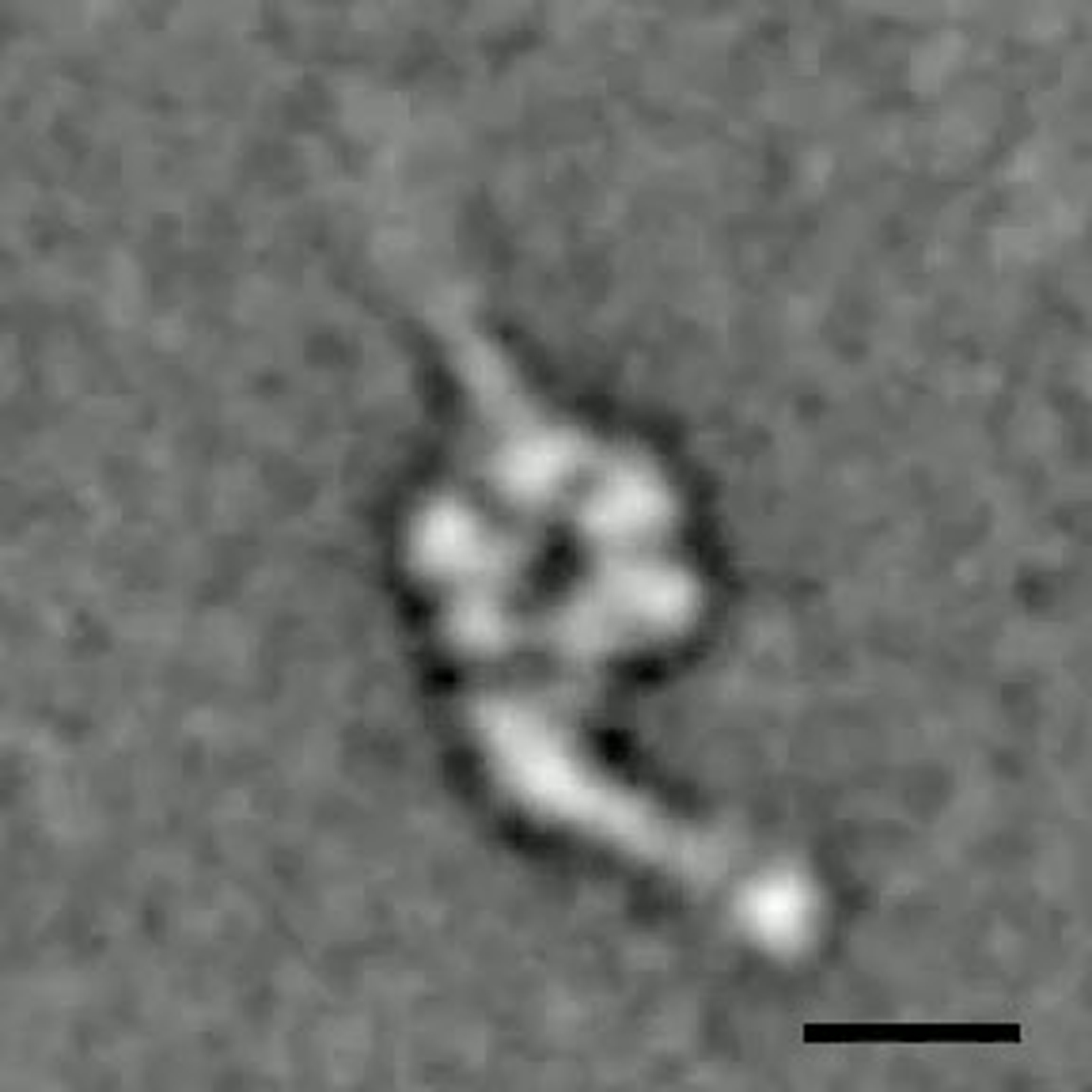

Supplement: Movie S1. Sequence of Class Averages Showing the Undocked Linker in the Motor-yC Construct, Related to Figure 5 [file mmc2.jpg]

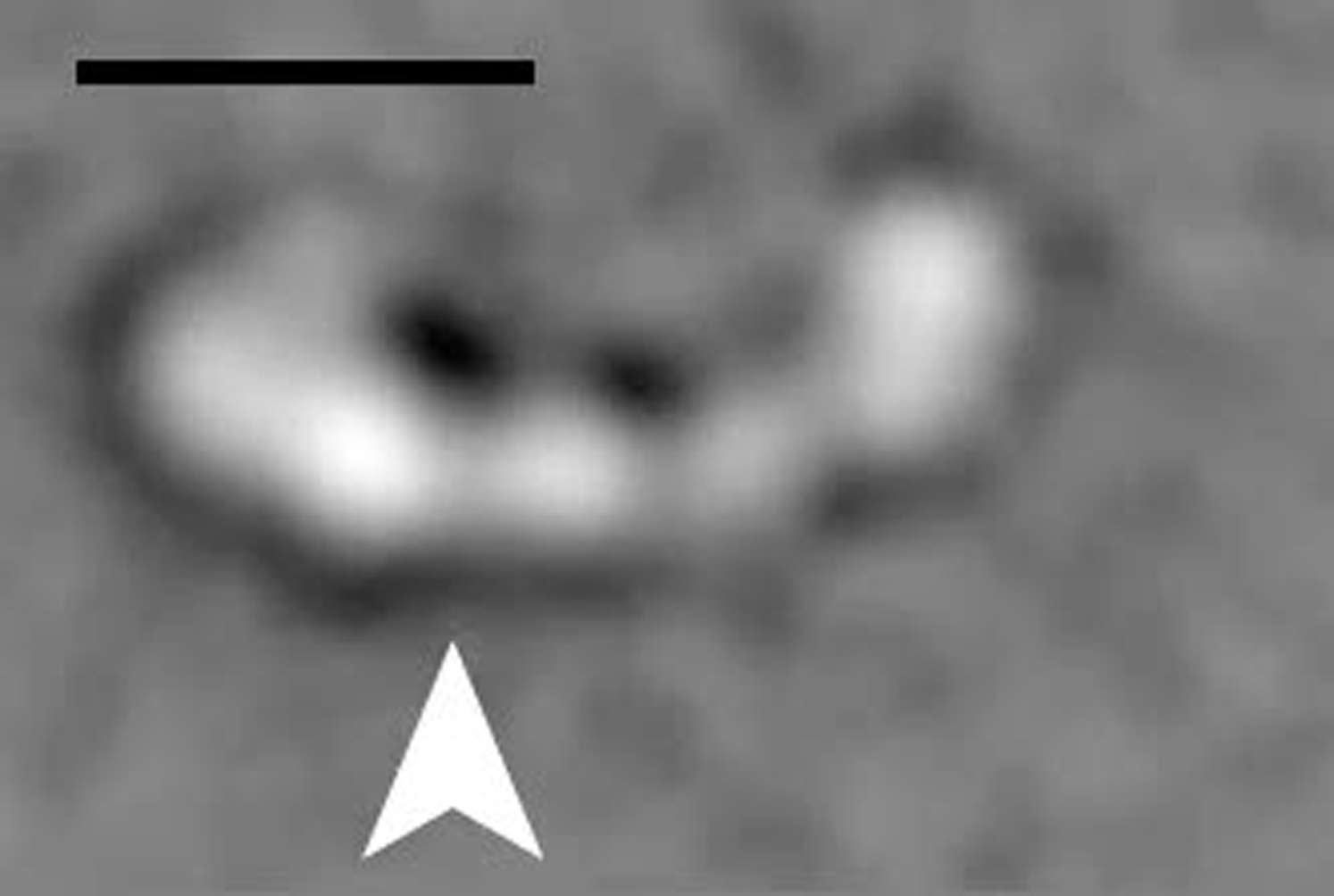

Supplement: Movie S2. Sequence of Linker Class Averages, following Alignment Based upon Undocked Linker Features, Related to Figure 5 [file mmc3.jpg]

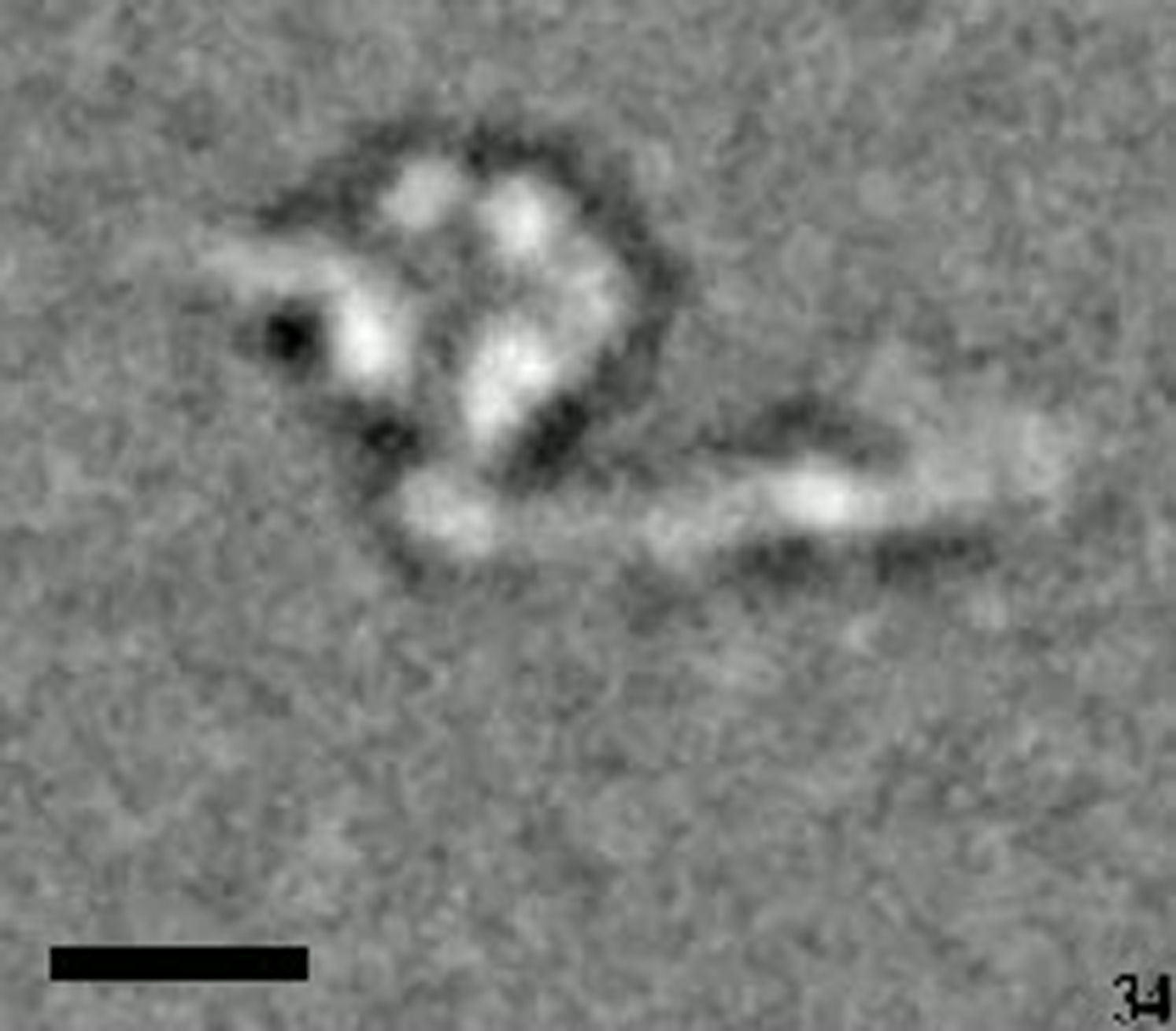

Supplement: Movie S3. Sequence of Cryo-EM Class Averages Showing Flexibility of the Tail Domain in Dynein-c, Related to Figure 6 [file mmc4.jpg]
